# Remote ischemic conditioning may improve graft function following kidney transplantation: a systematic review and meta-analysis with trial sequential analysis

**DOI:** 10.1186/s12871-024-02549-y

**Published:** 2024-05-03

**Authors:** Yang Zhang, Yuqin Long, Yongjun Li, Dawei Liao, Linkun Hu, Ke Peng, Hong Liu, Fuhai Ji, Xisheng Shan

**Affiliations:** 1 Department of Anesthesiology, Institute of Anesthesiology, The First Affiliated Hospital of Soochow University, Soochow University, Suzhou, Jiangsu China; 2 Institute of Anesthesiology, Soochow University, Suzhou, Jiangsu China; 3 Department of Anesthesiology, Zhongshan Hospital, Fudan University, Shanghai, China; 4 Department of Anesthesiology, Lianshui County People’s Hospital, Huaian, China; 5 Department of Anesthesiology, Tongren People’s Hospital, Tongren, Guizhou China; 6 Department of Neurology, The First Affiliated Hospital of Soochow University, Suzhou, China; 7 Department of Anesthesiology and Pain Medicine, University of California Davis Health, Sacramento, CA USA

**Keywords:** Remote ischemic conditioning, Kidney transplantation, Graft function, Systematic review

## Abstract

**Background:**

Remote ischemic conditioning (RIC) has the potential to benefit graft function following kidney transplantation by reducing ischemia-reperfusion injury; however, the current clinical evidence is inconclusive. This meta-analysis with trial sequential analysis (TSA) aimed to determine whether RIC improves graft function after kidney transplantation.

**Methods:**

A comprehensive search was conducted on PubMed, Cochrane Library, and EMBASE databases until June 20, 2023, to identify all randomized controlled trials that examined the impact of RIC on graft function after kidney transplantation. The primary outcome was the incidence of delayed graft function (DGF) post-kidney transplantation. The secondary outcomes included the incidence of acute rejection, graft loss, 3- and 12-month estimated glomerular filtration rates (eGFR), and the length of hospital stay. Subgroup analyses were conducted based on RIC procedures (preconditioning, perconditioning, or postconditioning), implementation sites (upper or lower extremity), and graft source (living or deceased donor).

**Results:**

Our meta-analysis included eight trials involving 1038 patients. Compared with the control, RIC did not significantly reduce the incidence of DGF (8.8% vs. 15.3%; risk ratio = 0.76, 95% confidence interval [CI], 0.48–1.21, *P* = 0.25, *I*^2^ = 16%), and TSA results showed that the required information size was not reached. However, the RIC group had a significantly increased eGFR at 3 months after transplantation (mean difference = 2.74 ml/min/1.73 m^2^, 95% CI: 1.44–4.05 ml/min/1.73 m^2^, *P <* 0.0001, *I*^2^ = 0%), with a sufficient evidence suggested by TSA. The secondary outcomes were comparable between the other secondary outcomes. The treatment effect of RIC did not differ between the subgroup analyses.

**Conclusion:**

In this meta-analysis with trial sequential analysis, RIC did not lead to a significant reduction in the incidence of DGF after kidney transplantation. Nonetheless, RIC demonstrated a positive correlation with 3-month eGFR. Given the limited number of patients included in this study, well-designed clinical trials with large sample sizes are required to validate the renoprotective benefits of RIC.

**Trial registration:**

This systematic review and meta-analysis was registered at the International Prospective Register of Systematic Reviews (Number CRD42023464447).

**Supplementary Information:**

The online version contains supplementary material available at 10.1186/s12871-024-02549-y.

## Introduction

Kidney transplantation is the treatment of choice for patients with end-stage renal disease [1, 2]. Every transplanted kidney inevitably undergoes ischemia following the loss of blood supply, which persists until reperfusion occurs after blood flow to the transplanted kidney is restored [3]. Ischemia/reperfusion (I/R) injury is a complex multifactorial pathophysiological process. I/R injury impairs the function of transplanted kidneys in the early postoperative period and is associated with an increased risk of delayed graft function (DGF), acute and chronic rejection, and graft failure [4, 5]. Given extremely limited donor resources, it is important to mitigate I/R injury and improve graft function [6, 7].

Remote ischemic conditioning (RIC) involves the implementation of brief and repetitive cycles of I/R in the extremities, inducing systemic protection against I/R injuries in distant organs. Both experimental and clinical studies have demonstrated its protective effects against I/R injury in various target organs such as the heart and kidney [8–10]. Since a study conducted in a porcine model of kidney transplantation first revealed that RIC provided benefits in terms of postoperative glomerular filtration rate and renal function, the effects of RIC in kidney transplantation have been increasingly explored in clinical settings [11]. Based on the timing of target organ ischemia, RIC can be classified into three types: remote ischemic preconditioning (RIPC, induced in the donor prior to target organ ischemia), remote ischemic perconditioning (RIPeC, induced in the recipient during target organ ischemia but before reperfusion), and remote ischemic postconditioning (RIPoC, induced in the recipient at the initiation of reperfusion) [12–14]. Considering feasibility, the former two approaches are by far the most commonly used interventions for kidney transplantation. However, the most effective conditioning strategy has yet to be established.

Owing to its easy-to-implement and non-invasive properties, RIC is an emerging and promising preventive measure for attenuating I/R injury in the perioperative period [3]. Although RIC has been documented as a renoprotective agent in animal models and clinical studies, its clinical benefits in kidney transplantation have not yet been fully harmonized in previous studies. A meta-analysis published in 2017 by Zhou et al. suggested that RIC does not improve graft function after kidney transplantation [15]. With the addition of newly published studies, the clinical effects may be further updated. Therefore, we designed this systematic review and meta-analysis of randomized controlled trials (RCTs) using trial sequential analysis (TSA) to evaluate the effects of RIC on DGF, acute rejection (AR), graft loss, estimated glomerular filtration rate (eGFR) at 3 or 12 months, and length of stay after kidney transplantation. We also explored the effects of each RIC approach on DGF in the subgroup analysis.

## Methods

### Protocol and registration

This systematic review and meta-analysis was registered in the International Prospective Register of Systematic Reviews (CRD42023464447) [16]. This study was conducted in accordance with the Preferred Reporting Items for Systematic Reviews and Meta-Analyses (PRISMA) guidelines [17]. The Preferred Reporting Items for Systematic Reviews and Meta-Analyses Checklist is presented in Supplementary Table 1.

### Search strategy

Three independent reviewers comprehensively searched the PubMed, EMBASE, and Cochrane Library databases using Medical Subject Headings and free text words, without any language restrictions. The final search was conducted on June 20, 2023. The detailed search strategy is presented in Supplementary Table 2. To ensure that all potentially eligible and relevant articles were included in this study, three reviewers conducted a manual search for possible bibliographies. The search results were imported and managed using the EndNote software (version 20.4, Thomson Reuters).

### Trial selection

The inclusion criteria for this meta-analysis were as follows: [1] study design: RCT; [2] participants: adults aged 18 years and above; [3] procedures: living or deceased donor kidney transplantation; and [4] studies with sufficient data available to evaluate short- or long-term outcomes, such as DGF, eGFR, AR, graft loss, and hospital stay duration. The exclusion criteria were as follows: [1] duplicate articles [2], studies that did not have specific outcomes, or [3] retrospective analyses, case reports, meeting abstracts, and trial protocols.

Three reviewers independently screened the titles and abstracts of each paper and read the full text of potentially eligible studies. If discrepancies occurred during trial selection, the three reviewers collaborated to achieve resolution.

### Data extraction

Two reviewers independently extracted the following information from each included RCT: the first author’s name, year of publication, comparison groups, number of patients, interventions in the control group, and study outcomes. In case of any discrepancies during data extraction, the two reviewers collaborated to achieve a consensus.

### Primary outcome

The primary outcome was the incidence of DGF, which was defined as the need for dialysis during the first post-transplant week.

### Secondary outcome

The prespecified secondary outcomes were the incidence of AR, graft loss, length of hospital stay, and eGFR at 3 and 12 months. AR was diagnosed based on biopsy of the kidney graft. Graft loss was defined as return to regular dialysis or graft removal. The eGFR was calculated based on serum creatinine levels using established Eq. (18,19). Three studies used the Modification of Diet in Renal Disease (MDRD) formula to calculate eGFR [20–22], while four studies used the Chronic Kidney Disease Epidemiology Collaboration (CKD-EPI) equation to calculate eGFR [23–26].

### Quality assessment

Two reviewers used the Cochrane Collaboration tool to evaluate the quality of the included studies [27]. This tool comprises seven distinct sections that cover various techniques to minimize bias. These sections included generating randomized sequences, concealing allocations, ensuring blindness among participants and staff, evaluating results without bias, addressing inadequate outcome data, avoiding selective reporting, and identifying additional sources of bias. Each study was assessed for the risk of bias and assigned one of the following three ratings: high (indicating a high risk in one or more categories), low (signifying a low risk in all domains), or unclear. Any discrepancies between the two reviewers’ assessments were resolved by discussion.

### Statistical analysis

For dichotomous and continuous variables, treatment effects were assessed using the risk ratio (RR) and weighted mean difference (WMD), with their corresponding 95% confidence intervals (CI). Subgroup analyses based on RIC type, implementation sites, and graft donor categories were also conducted. The statistical significance of RR and WMD was determined using the Z-test, and P values less than 0.05 were deemed significant. For the five pre-defined secondary outcomes, multiple testing correction was employed using the Bonferroni method, with *P* < 0.01 indicating statistical significance (i.e., 0.05/5). Because of the heterogeneity of clinical studies, we used a random-effects model for data polling [28]. The I^2^ statistic was used to measure heterogeneity, and an I^2^ value of greater than 50% indicated significant heterogeneity [29, 30]. Subsequently, we plotted Begg’s funnel plot and used Egger’s test to assess publication bias [31, 32]. All statistical analyses were conducted using Review Manager (version 5.4; Cochrane Collaboration, Oxford, UK).

To assess whether the evidence in the meta-analysis was reliable and sufficient to detect an effect, we applied TSA viewer software (version 0.9.5.5 beta, Copenhagen Trial Unit, Centre for Clinical Intervention Research, Rigshospitalet, Copenhagen, Denmark) [33, 34]. In a TSA diagram, a Z-curve crossing the trial sequence monitoring boundary or futility boundary denotes that the evidence is currently adequate to draw a conclusion and that additional research is unlikely to alter the inference. By contrast, if the Z-curve does not cross any of the boundaries, the evidence is insufficient. D^2^ (diversity) was defined as heterogeneity correction. Considering a previous meta-analysis [35], we used a type 1 error of 5%, a power of 80%, and two-sided testing to conduct this analysis for dichotomous variables. Kim et al. applied RIC in living donor kidney transplantation and showed that the incidence of DGF was reduced from 30 to 20%, that is a relative risk reduction (RRR) of 33%. Based on this data, we set a consistent RRR value in our study to 30% [22]. For continuous variables, a type 1 error of 5%, power of 90%, and two-sided tests were set up to calculate the required information size, mean difference, and variance based on empirical assumptions, which were autogenously generated by software [36]. Variance-based O’Brien-Fleming heterogeneity correction and O’Brien-Fleming alpha and beta spending functions were utilized.

## Results

### Study characteristics

**Fig. 1:**
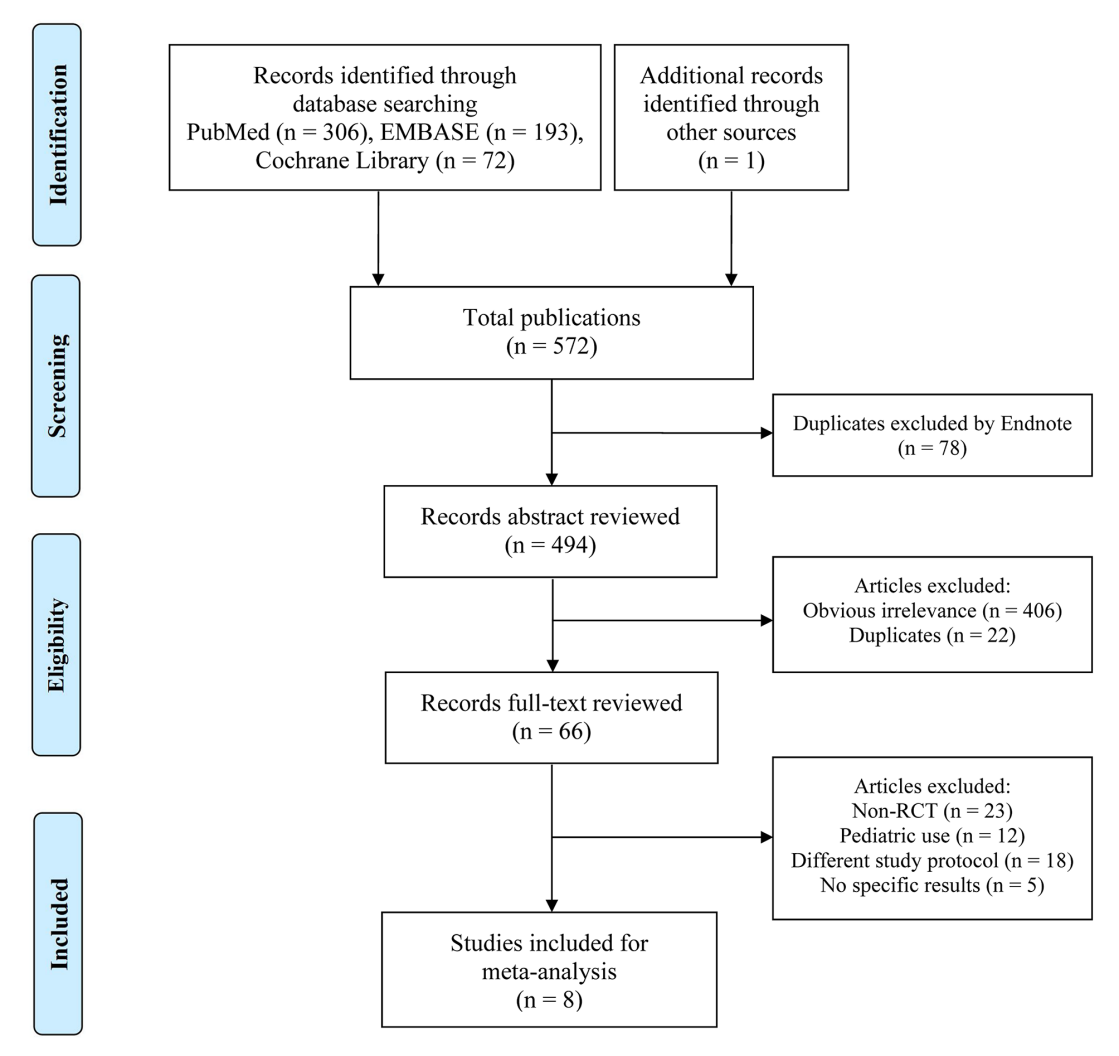
Flow diagram of study selection process

**Fig. 2:**
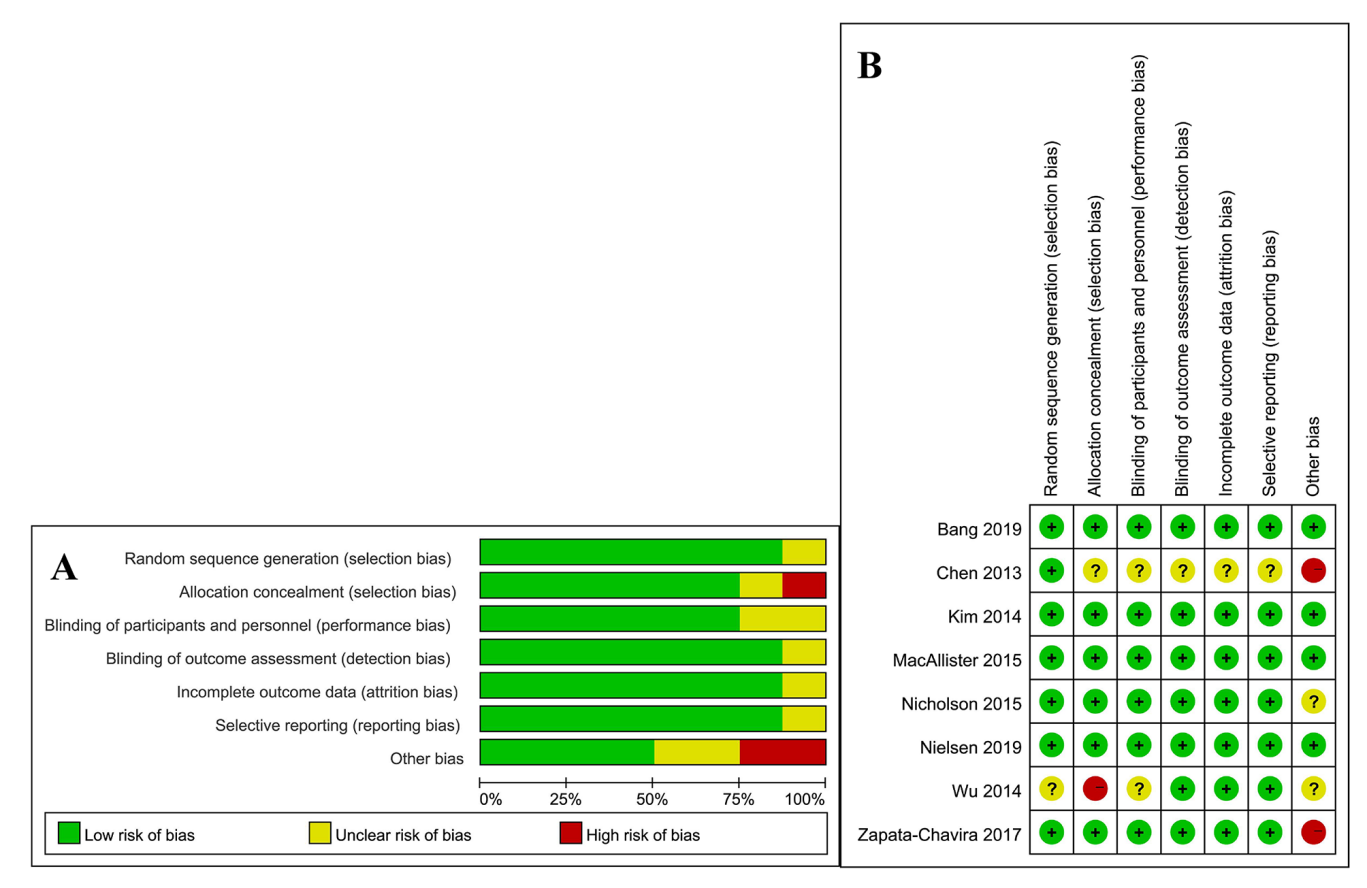
Risk of bias assessment. (**A**) risk of bias graph; (**B**) risk of bias summary

The initial literature search yielded a total of 572 articles. Among them, eight were finally included [20–26, 37](Fig. 1). The characteristics of the eight studies that involved 1038 participants, are summarized in Table 1. The RIC protocols in the included RCTs were not entirely consistent: preconditioning in 3 trials [24, 25, 37], perconditioning in 3 trials [20, 21, 23], postconditioning in 1 trial [22], and combined preconditioning and perconditioning in 1 trial [26]. Four trials used three or four cycles of 5-minute ischemia (200–300 mmHg of inflation pressure, or 25 mmHg more than the systolic blood pressure) and 5-minute reperfusion in the thigh [20, 23, 25, 37], Three trials employed three or four cycles of 5-minute ischemia (200 mmHg of inflation pressure, or 40 mmHg more than the systolic blood pressure) and 5-minute reperfusion in the upper arm [22, 24, 26], the other trials performed three cycles of 5-minute ischemia and 5-minute reperfusion in the iliac artery without details of the pressure [21]. In each study, RIC was compared with a sham process (inflation pressure less than 20 mm Hg or a deflated cuff). Of the eight trials, five were performed for living donor kidney transplants [20, 22, 24, 26, 37] and three were performed for deceased donor kidney transplants [21, 23, 25]. Three trials were assessed to be at high risk, Chen and Zapata-Chavira’s, due to a sample size of less than 40 cases, while Wu’s was due to a lack of randomization [21, 25, 37](Fig. 2A and B).

**Table 1:** Characteristics of the included studies

Author	Year	Group: *n*	RIC type	RIC protocol	Control	Donor type	Outcomes reported
Nielsen	2019	RIC: 109 Control: 113	RIPeC	Thigh, 4*5 min cycle	Sham procedure	DCD/DBD	Death, On dialysis, Rejection, DGF, eGFR, sCr, cys C and NGAL 3/12months
Bang	2019	RIC: 85 Control: 85	RIPC	Upper arm, 3*5 min cycles, 200 mmHg	Sham procedure	Living-donor	eGFR 3/12/24/36/48/60 months, DGF, Graft loss, Death and Graft loss or death 5 years
Zapata-Chavira	2017	RIC: 17 Control: 12	RIPC	Thigh, 1*10 min cycle, 200 mmHg	Sham procedure	DCD/DBD	sCr、BUN and eGFR 12/24/48/72 h, 7/15/30/90 days
Nicholson	2015	RIC: 40 Control: 40	RIPeC	Thigh, 4*5 min cycle, 200 mm Hg or 25 mmHg above systolic pressure	Sham procedure	Living-donor	sCr and eGFR 1/3months, DGF
MacAllister	2015	RIC: 307 Control: 99	RIPC + RIPeC	Upper arm, 4*5 min cycles, 40 mmHg above systolic pressure	Sham procedure	Living-donor	hospital stay, eGFR 3/12 months, DGF; AR, Graft loss, death 1 year
Wu	2014	RIC: 24 Control: 24	RIPeC	The iliac artery, 3*5 min cycles	Sham procedure	DCD	DGF; sCr, eGFR 1/2/3/4/5/6/7/14/30 days; NGAL 2/12/24/48 h
Kim	2014	RIC: 30 Control: 30	RIPoC	Upper arm, 3*5 min cycles	Sham procedure	Living-donor	sCr and eGFR 1 year, DGF, hospital stay
Chen	2013	RIC: 20 Control: 20	RIPC	Thigh, 3*5 min cycle, 300 mmHg	Sham procedure	Living-donor	DGF; hospital stay; Urine volume; sCr 1/4/24/48/72 h and 7/14 days; NGAL 1/4/24 h

RIC, Remote Ischemic Conditioning; RIPC, Remote Ischemic Preconditioning; RIPeC, Remote Ischemic Perconditioning; RIPoC, Remote Ischemic Postconditioning; DGF, delayed graft function; DCD, donation after cardiac death; DBD, donation after brain death; eGFR, estimated glomerular filtration rate; sCr, serum creatinine; cys C, cystatin C; NGAL, neutrophil gelatinase-associated lipocalin; BUN, blood urea nitrogen; AR, Acute Rejection; tCr50, a 50% decrease in baseline plasma creatinine

### Effects of RIC on the primary outcome

**Fig. 3:**
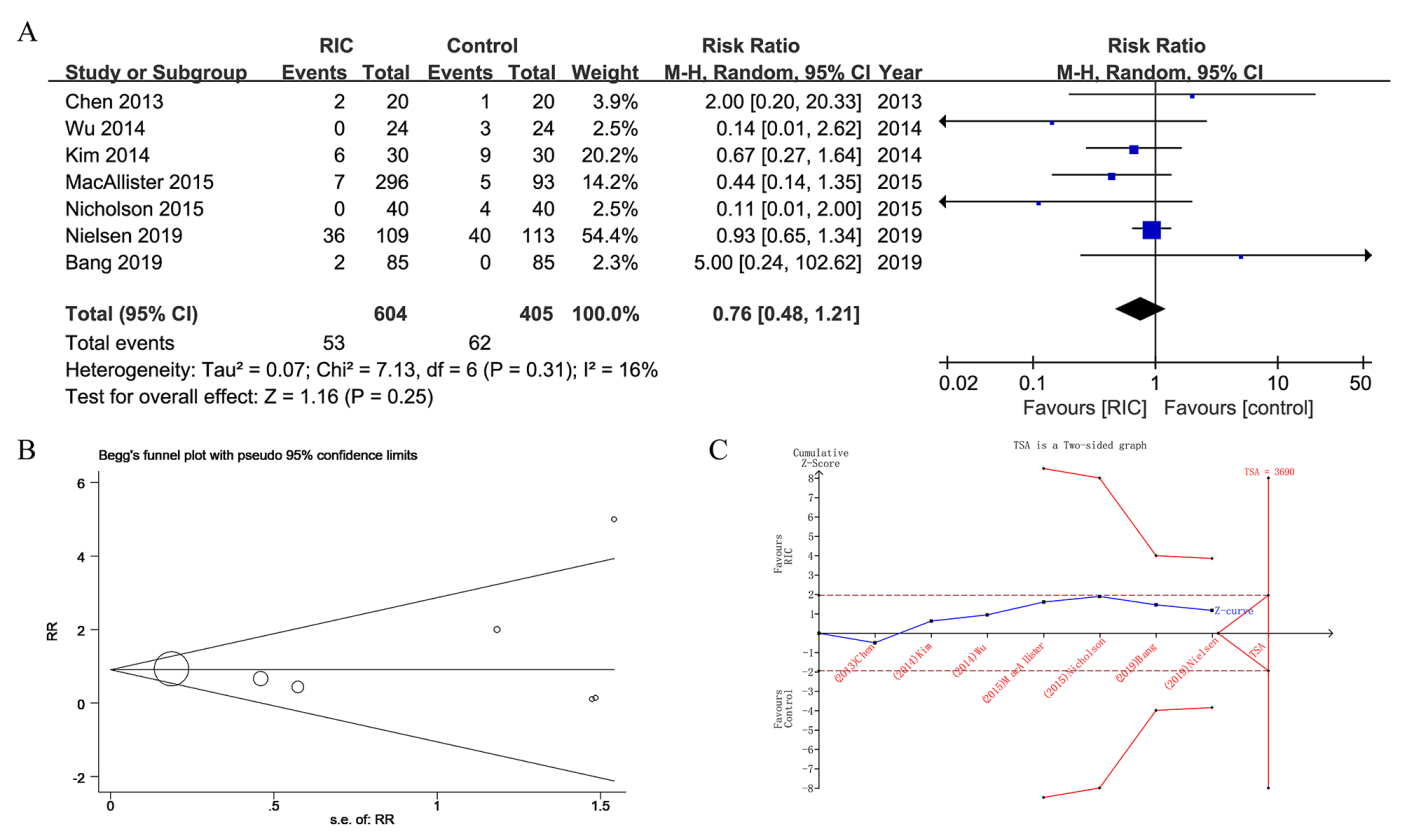
Pooled result of DGF in patients undergoing kidney transplantation between RIC and control. (**A**) forest plot; (**B**) Begg’s funnel plot; (**C**) TSA diagram. TSA analysis is based on a relative risk reduction (RRR) of 30% and a control event rate of 15.3%. The inward sloping red lines indicate the trial sequential monitoring boundary, the outward sloping red lines indicate the futility boundary; brown lines indicate the conventional benefit boundary; blue line is the Z-curve; SD, standard deviation; CI, confidence interval; M-H, Mantel-Haenszel; RIC, remote ischemic conditioning; RR, risk ratio; s.e., standard error; TSA, Trial sequential analysis; RIS, Required information size

The effects of RIC on postoperative outcomes are summarized in Table 2. Seven studies including 1009 patients reported the incidence of DGF [20–24, 26, 37]. Compared to the control group, the RIC group did not show a significant difference in the incidence of DGF (8.8% vs. 15.3%; RR = 0.76, 95% CI, 0.48–1.21, *P* = 0.25, *I*^2^  = 16%) (Fig. 3A). There was no publication bias in Begg’s funnel plot (*P* = 0.368, Fig. 3B) or Egger’s test (*P =* 0.599). In the TSA analysis (Fig. 3C), with the assumption of a 30% RRR in the incidence of DGF from 15.3% in the control group to 10.7% in the RIC group, the Z-curve (blue) crossed neither the conventional benefit boundary (brown) nor the trial sequential monitoring boundary (red). The required sample size for DGF was estimated to be 3690, while the accrued sample size in this analysis was 1009. The results of the TSA suggested that there was insufficient evidence from existing meta-analyses to make a definitive conclusion.

**Table 2:** Summary of outcomes

Outcomes	RIC(*n*)	Control(*n*)	Effect size (95% CI)	*P* value	I² (%)
Primary outcomes					
DGF	53/604	62/405	RR = 0.76 (0.48 to 1.21)	0.25	16
**Secondary outcomes**					
AR	67/595	39/389	RR = 1.11 (0.76 to 1.64)	0.58	0
Graft loss	15/565	15/357	RR = 0.76 (0.38 to 1.51)	0.43	0
eGFR 3 m, ml min^− 1^(1.73 m)^−2^	455	269	MD = 2.74 (1.44 to 4.05)	< 0.001	0
eGFR 12 m, ml min^− 1^(1.73 m)^−2^	484	303	MD = 1.98 (-0.74 to 4.70)	0.15	0
Hospital stay, d	199	203	MD =-0.73 (-1.56 to 0.11)	0.09	0

RIC, Remote Ischemic Conditioning; eGFR, estimated glomerular filtration rate; MD, mean difference; DGF, delayed graft function; RR, risk ratio; AR, Acute Rejection; CI, confidence interval

### Effects of RIC on the secondary outcomes

#### Incidence of AR, graft loss, length of hospital stay

Six studies including 984 patients reported AR after transplantation, with an incidence ranging from 0 to 17.5% (Table 2 and Supplementary Fig. 1). No significant difference was observed in the incidence of AR between the two groups (RIC vs. Control: RR = 1.11, 95% CI, 0.76–1.64, *P* = 0.58, *I*^2^ = 0%). In the TSA analysis, the Z-curve (blue) crossed neither the conventional benefit boundary (brown) nor the trial sequential monitoring boundary (red) for AR.

Five studies including 922 patients presented data of graft loss within 12 months after transplantation (Table 2 and Supplementary Fig. 2), and there was no between-group difference (RIC vs. Control groups: RR = 0.76, 95% CI, 0.38–1.51, *P* = 0.43, *I*^2^ = 0%). TSA indicated that the Z-curve (blue) crossed neither the conventional benefit boundary (brown) nor the trial sequential monitoring boundary (red) in relation to graft loss.

With respect to the length of hospital stay (Table 2 and Supplementary Fig. 3), no significant difference (RIC vs. control groups: MD= -0.73, 95% CI: -1.56-0.11, *P =* 0.09, *I*^2^ = 0%) was found after collecting pooled data from the four studies. For the length of hospital stay, the required information size was not reached according to TSA analysis.

#### eGFR at 3 months and 12 months

**Fig. 4:**
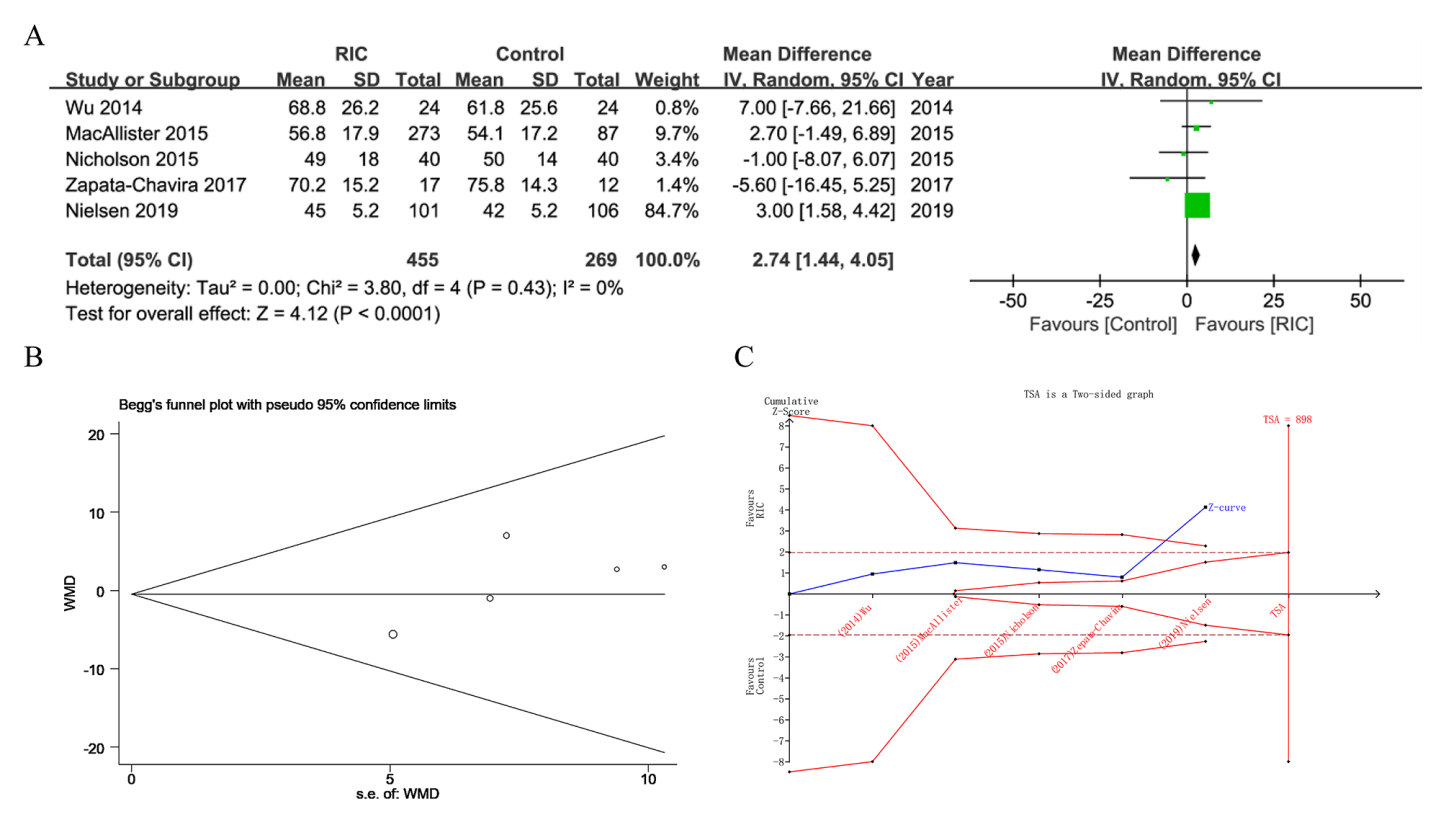
Pooled result of eGFR at 3 months in patients undergoing kidney transplantation between RIC and control. (**A**) forest plot; (**B**) Begg’s funnel plot; (**C**) TSA diagram. Analysis is based on a power of 90%. The inward sloping red lines indicate the trial sequential monitoring boundary, the outward sloping red lines indicate the futility boundary; brown lines indicate the conventional benefit boundary; blue line is the Z-curve; SD, standard deviation; CI, confidence interval; M-H, Mantel-Haenszel; RIC, remote ischemic conditioning; WMD, weighted mean difference; s.e., standard error; TSA, Trial sequential analysis; RIS, Required information size

Five studies, including 724 patients, presented eGFR results 3 months after transplantation (Table 2; Fig. 4). 3-month eGFR was significantly higher in the RIC group than that in the control group (RIC vs. Control: MD = 2.74 ml/min/1.73 m^2^, 95% CI: 1.44–4.05 ml/min/1.73 m^2^, *P* < 0.0001, *I*^2^ = 0%), even after multiple test corrections. The TSA diagram indicated that the Z-curve for eGFR at 3 months crossed both the trial sequential monitoring boundary (red) and conventional benefit boundary (brown), suggesting sufficient evidence for this result. eGFR at 12 months was documented in 4 studies (Table 2 and Supplementary Fig. 4), and no significant difference was found between groups (RIC vs. Control: MD = 1.98 ml/min/1.73 m^2^, 95% CI: -0.74-4.70 ml/min/1.73 m^2^, *P =* 0.15, *I*^2^ = 0%). The required information size for the 12-month eGFR was not reached, based on TSA analysis.

### Subgroup analyses for DGF

To delve deeper into the factors potentially impacting DGF, we undertook subgroup analyses from three distinct perspectives and present the findings of our research (Table 3). Subgroup analysis based on RIC type (RIPC vs. RIPeC with or without RIPC vs. RIPoC) showed no interaction among the groups in the incidence of DGF (Supplementary Fig. 5). In the subgroup analyses (upper arm vs. thigh), neither location site in the RIC group significantly improved the incidence of DGF after kidney transplantation (Supplementary Fig. 6). Furthermore, regarding the classification of living and deceased donors, the RIC group did not show superiority in either of the categories (Supplementary Fig. 7).

**Table 3:** Subgroup analyses for DGF

DGF	RIC (*n*)	Control (*n*)	RR (95% CI)	*P* value	I²(%)	*P* interaction
RIPC	4/105	1/105	2.81 (0.45 to 17.68)	0.27	0	0.29
RIPeC with or without RIPC	43/469	52/270	0.55 (0.24 to 1.29)	0.17	42	
RIPoC	6/30	9/30	0.67 (0.27 to 1.64)	0.38		
upper arm	15/411	14/208	0.64 (0.30 to 1.38)	0.26	12	0.65
thigh	38/169	45/173	0.85 (0.34 to 2.14)	0.73	22	
living donor	17/471	19/268	0.65 (0.31 to 1.34)	0.24	12	0.99
deceased donor	36/133	43/137	0.64 (0.14 to 2.87)	0.56	38	

RIC, Remote Ischemic Conditioning; RIPC, Remote Ischemic Preconditioning; RIPeC, Remote Ischemic Perconditioning; RIPoC, Remote Ischemic Postconditioning; CI, confidence interval

### Subgroup analyses for secondary outcomes

In the subgroup analysis based on RIC type (RIPC vs. RIPeC with or without RIPC vs. RIPoC), there were no subgroup differences with regard to AR, graft loss, length of hospital stay, or eGFR at 3 and 12 months (Supplementary Figs. 8–12). In subgroup analyses based on upper arm vs. thigh and living donor vs. deceased donor, no subgroup differences were detected in relation to AR, graft loss, length of hospital stay, or eGFR at 3 and 12 months (Supplementary Figs. 13–22).

## Discussion

This meta-analysis investigated the potential renal benefits of RIC in kidney patients. Eight RCTs with 1038 patients were included in this study. The RIC group appeared to have a lower incidence of DGF than the control group (8.8% vs. 15.3%), but the difference was not statistically significant. The results of the TSA analysis suggest that the current sample size was inadequate to make a definitive conclusion. Furthermore, the results of this study demonstrated that the RIC group had a significantly higher eGFR at three months than the control group, even after multiple testing corrections. Taken together, the current results suggest that RIC procedures could provide a certain extent of nephroprotection in patients undergoing kidney transplantation.

RIC is a safe, non-invasive, and nonpharmacological therapy to mitigate I/R injury and involves several brief cycles of ischemia and reperfusion of an organ or tissue (such as using a blood pressure cuff on the limb). Protection against I/R injury by transient ischemia at sites remote from the target organ in dogs was first described in 1993 by Przyklenk et al., and this concept has rapidly developed in recent years [38]. In various clinical fields, this intervention has been applied to a wide range of organs, including the heart, brain, and kidneys [39–42]. The mechanisms underlying the protective effects of RIC have been explored extensively, but have not been fully clarified. Nonetheless, it has been suggested that a protective signal is generated at a distant site and transmitted to target organs through generalized humoral, neural, and systemic generalized responses [9]. Several trigger factors (such as autacoids, endocannabinoids, stromal cell-derived factor-1α, and miR-144) are induced by the RIC charge in the transmission of the signal from the conditioned tissue to the target organ [43–45]. Through the activation of a series of intracellular signaling pathways, including the reperfusion injury salvage kinase (RISK) pathway, cyclic guanosine monophosphate/protein kinase C (cGMP/PKC) pathway, and survivor activating factor enhancement (SAFE) pathway, the signal is finally passed to the effectors, identified as mitochondria or downstream molecules. These effects protect cells from mitochondrial dysfunction, microvascular endothelial dysfunction, oxidative stress, inflammation, and apoptosis, thereby suppressing I/R injury [46].

RIC has evolved into a promising strategy for nephroprotection, and has been documented in several clinical studies. To date, favorable results on the renoprotective effect of RIC have been reported mainly in cardiovascular procedures [10, 47, 48]. Although its nephroprotective effect in kidney transplantation has been confirmed in large-animal models [11, 49], randomized controlled studies examining the role of RIC in renal transplantation are still underway [50]. A previous meta-analysis published in 2017, including 651 recipients in six studies, showed that RIC did not contribute to any improvements in graft function after kidney transplantation. Stratified analysis based on RIC type also failed to draw definitive conclusions. The unfavorable results of this meta-analysis can be attributed to the inadequate sample size. In our meta-analysis, we included two recently published articles and performed TSA on the main graft function. As a result, we achieved favorable results in the secondary outcome of 3-month eGFR, and firm evidence was suggested for this outcome in TSA analysis. In addition, subgroup analyses of RIC type, implementation sites, and graft source were features of our meta-analysis, suggesting that perconditioning with or without preconditioning is more likely to improve graft function. Another recently published meta-analysis including 11 studies with 1145 patients showed that RIC could reduce serum creatinine levels in the early postoperative period and improve eGFR 3 months after surgery [50]. These results were consistent with our findings. However, that meta-analysis conflated kidney transplantation and partial nephrectomy, which were completely different surgical procedures with different mechanisms of renal ischemia reperfusion. Our research, in contrast, exclusively focused on kidney transplants. Furthermore, that meta-analysis confined its subgroup analysis solely to these procedures, without exploring the details of RIC, such as different RIC types, application sites, and donor types. Our study not only performed these subgroup analyses but also incorporated TSA to ascertain the required information size. Consequently, our results are more convincing and clinically relevant.

The effectiveness of RIC depends on the protocol used. A range of protocols, including RIPC, RIPeC, and RIPoC, have been employed in clinical settings; however, there is no consensus in defining the most favorable protocol. Studies have suggested that the timing and duration of RIC stimuli seem to have protective effects [51–53]. Considering the complex allotransplantation procedure, the graft is transferred from the donor to the recipient after perfusion and cold preservation. Therefore, unilateral preconditioning of the donor does not protect the graft throughout the entire process [23, 54]. It is also believed that protective humoral factors released from the donor’s conditioned tissues are no longer in circulation at the time of reperfusion. Hence, application of RIC to the recipient (RIPeC or RIPoC) or in combination with RIPC may produce more stable effects. Among previous clinical studies, only Macallister et al. implemented combined RIPeC and RIPC procedures on recipients and donors simultaneously in living kidney transplants, and their study ultimately achieved favorable results in terms of improved postoperative eGFR [26]. In our subgroup analysis for eGFR at 3 months, RIPeC with or without RIPC appeared to be more beneficial to recipients than the other RIC procedures. Therefore, more randomized controlled clinical studies should be conducted to confirm the validity of the RIPeC or combined RIC approaches.

The volume of remote tissue that causes ischemia during conditioning influences the intensity of protection [37]. Considering the different muscle mass conditions, we compared the protective effect of RIC at different conditioned sites in the upper or lower extremities; however, we could not find a significant difference between the subgroups. Franchello et al. demonstrated that ischemic conditioning on marginal liver grafts provided better outcomes than conditioning on high-quality grafts, indicating that ischemic conditioning may provide more protection for poor-quality renal grafts [55]. Kim also reported that the inability to detect a favorable effect of RIC was attributed to the relatively small degree of ischemia-reperfusion injury in living-donor kidney transplants [22]. In our study, the majority of patients who underwent kidney transplantation received living donor kidneys. This is a lower risk group with a low incidence of DGF. It could be one possible reason for not observing significant benefit from RIC. Therefore, future studies should aim to evaluate potential advantages of RIC in kidney transplant recipients at higher risk of DGF and poorer prognoses, such as those receiving kidneys from older donors, kidneys with extended cold ischemia time, and kidneys from donors after cardiac death.

In contrast to simple animal experimental settings, several confounding factors in clinical studies may have compromised our findings. Many studies have proposed that comorbidities and the concomitant use of medications, immunosuppressive drugs, and anesthetics can interfere with RIC-induced protection [13, 56, 57]. Previous clinical studies have illustrated that older age and comorbidities including hyperlipidemia, diabetes, and hypertension, elevated the threshold for protection, and more robust conditioning signals are required [13]. Sevoflurane and desflurane were the principal anesthetic agents used in the included trials. Volatile anesthetics have been reported to imitate the early stages of ischemic preconditioning via multi-pathway signaling of mitochondrial K_ATP_ channels, which may interfere with the protective effects of RIC [58, 59].

There are several limitations that need to be addressed. First, the TSA suggests a relatively small sample size in the included studies, which may have reduced statistical power. Second, the majority of participants in the studies were living donor transplants, as reflected in the very low rates of DGF observed in both groups. However, DGF has been documented as an important indicator in almost all the studies. Third, the postoperative follow-up period was up to one year, we did not have data on long-term renal function. Fourth, more sensitive indicators of renal injury were not identified in previous studies. Fifth, a relatively small sample size was used in the subgroup analysis.

## Conclusion

In this meta-analysis with trial sequential analysis, RIC did not lead to a significant reduction in the incidence of DGF after kidney transplantation. Nonetheless, RIC demonstrated a positive correlation with 3-month eGFR. Given the limited number of patients included in this study, well-designed clinical trials with large sample sizes are required to validate the renoprotective benefits of RIC.

### Electronic supplementary material

Below is the link to the electronic supplementary material.

Supplementary Material 1

## Data Availability

No datasets were generated or analysed during the current study.
