# Supplementary material for: Remote ischemic conditioning may improve graft function following kidney transplantation: a systematic review and meta-analysis with trial sequential analysis
Source: BMC Anesthesiol. 2024 May 3;24:168. doi: 10.1186/s12871-024-02549-y (PMC11067269; doi:10.1186/s12871-024-02549-y)
Supplement: Supplementary file 1 — Supplementary Material 1 [file 12871_2024_2549_MOESM1_ESM.docx]

Supplementary Material

**Effect of Remote Ischemic Conditioning on Graft Function Following Kidney Transplantation: A Systematic Review With Meta-analysis and Trial Sequential Analysis**

**Supplementary Table 1. PRISMA checklist**

| **Section/topic** | **#** | **Checklist item** | **Reported on page #** |
| --- | --- | --- | --- |
| **TITLE** | | |  |
| Title | 1 | Identify the report as a systematic review, meta-analysis, or both. | 1 |
| **ABSTRACT** | | |  |
| Structured summary | 2 | Provide a structured summary including, as applicable: background; objectives; data sources; study eligibility criteria, participants, and interventions; study appraisal and synthesis methods; results; limitations; conclusions and implications of key findings; systematic review registration number. | 1 |
| **INTRODUCTION** | | |  |
| Rationale | 3 | Describe the rationale for the review in the context of what is already known. | 2 |
| Objectives | 4 | Provide an explicit statement of questions being addressed with reference to participants, interventions, comparisons, outcomes, and study design (PICOS). | 2 |
| **METHODS** | | |  |
| Protocol and registration | 5 | Indicate if a review protocol exists, if and where it can be accessed (e.g., Web address), and, if available, provide registration information including registration number. | 2 |
| Eligibility criteria | 6 | Specify study characteristics (e.g., PICOS, length of follow-up) and report characteristics (e.g., years considered, language, publication status) used as criteria for eligibility, giving rationale. | 2-3 |
| Information sources | 7 | Describe all information sources (e.g., databases with dates of coverage, contact with study authors to identify additional studies) in the search and date last searched. | 2 |
| Search | 8 | Present full electronic search strategy for at least one database, including any limits used, such that it could be repeated. | 2 |
| Study selection | 9 | State the process for selecting studies (i.e., screening, eligibility, included in systematic review, and, if applicable, included in the meta-analysis). | 2-3 |
| Data collection process | 10 | Describe method of data extraction from reports (e.g., piloted forms, independently, in duplicate) and any processes for obtaining and confirming data from investigators. | 3 |
| Data items | 11 | List and define all variables for which data were sought (e.g., PICOS, funding sources) and any assumptions and simplifications made. | 3 |
| Risk of bias in individual studies | 12 | Describe methods used for assessing risk of bias of individual studies (including specification of whether this was done at the study or outcome level), and how this information is to be used in any data synthesis. | 3 |
| Summary measures | 13 | State the principal summary measures (e.g., risk ratio, difference in means). | 3 |
| Synthesis of results | 14 | Describe the methods of handling data and combining results of studies, if done, including measures of consistency (e.g., I^2^) for each meta-analysis. | 3 |
| Risk of bias across studies | 15 | Specify any assessment of risk of bias that may affect the cumulative evidence (e.g., publication bias, selective reporting within studies). | 3 |
| Additional analyses | 16 | Describe methods of additional analyses (e.g., sensitivity or subgroup analyses, meta-regression), if done, indicating which were pre-specified. | 3 |
| **RESULTS** | | |  |
| Study selection | 17 | Give numbers of studies screened, assessed for eligibility, and included in the review, with reasons for exclusions at each stage, ideally with a flow diagram. | 4 |
| Study characteristics | 18 | For each study, present characteristics for which data were extracted (e.g., study size, PICOS, follow-up period) and provide the citations. | 4 |
| Risk of bias within studies | 19 | Present data on risk of bias of each study and, if available, any outcome level assessment (see item 12). | 4 |
| Results of individual studies | 20 | For all outcomes considered (benefits or harms), present, for each study: (a) simple summary data for each intervention group (b) effect estimates and confidence intervals, ideally with a forest plot. | 5-6 |
| Synthesis of results | 21 | Present results of each meta-analysis done, including confidence intervals and measures of consistency. | 5-6 |
| Risk of bias across studies | 22 | Present results of any assessment of risk of bias across studies (see Item 15). | 5-6 |
| Additional analysis | 23 | Give results of additional analyses, if done (e.g., sensitivity or subgroup analyses, meta-regression [see Item 16]). | 6-7 |
| **DISCUSSION** | | |  |
| Summary of evidence | 24 | Summarize the main findings including the strength of evidence for each main outcome; consider their relevance to key groups (e.g., healthcare providers, users, and policy makers). | 7-9 |
| Limitations | 25 | Discuss limitations at study and outcome level (e.g., risk of bias), and at review-level (e.g., incomplete retrieval of identified research, reporting bias). | 9 |
| Conclusions | 26 | Provide a general interpretation of the results in the context of other evidence, and implications for future research. | 9 |
| **FUNDING** | | |  |
| Funding | 27 | Describe sources of funding for the systematic review and other support (e.g., supply of data); role of funders for the systematic review. | 10 |

**Supplementary Table 2.** **Search strategies**

**PubMed**

| **Search** | **Query** |
| --- | --- |
| #1 | "Ischemic Preconditioning" [mh] OR "Ischemic Postconditioning"[mh] |
| #2 | (preconditioning, ischemic) OR (ischemic pre-conditioning) OR (ischemic pre conditioning) OR (pre-conditioning, ischemic) OR (remote ischemic preconditioning) OR (ischaemic preconditioning) OR (remote ischaemic preconditioning) OR (RIPC) OR (Postconditioning, Ischemic) OR (Ischemic Post-Conditioning) OR (Ischemic Post Conditioning) OR (Post-Conditioning, Ischemic) OR (Ischemic conditioning) |
| #3 | #1 OR #2 |
| #4 | "Kidney Transplantation" [mh] |
| #5 | (Renal Transplantation) OR (Renal Transplantations) OR (Transplantations, Renal) OR (Transplantation, Renal) OR (Grafting, Kidney) OR (Kidney Grafting) OR (Transplantation, Kidney) OR (Kidney Transplantations) OR (Transplantations, Kidney) |
| #6 | #4 OR #5 |
| #7 | #3 AND #6 |

**EMBASE**

| **Search** | **Query** |
| --- | --- |
| #1 | 'Ischemic Preconditioning'/exp |
| #2 | 'ischemic preconditioning' OR 'preconditioning, ischemic' OR 'ischemic pre-conditioning' OR 'ischemic pre conditioning' OR 'pre-conditioning, ischemic' OR 'remote ischemic preconditioning'/exp OR 'remote ischemic preconditioning' OR 'ischaemic preconditioning'/exp OR 'ischaemic preconditioning' OR 'remote ischaemic preconditioning'/exp OR 'remote ischaemic preconditioning' OR 'ripc' OR 'postconditioning, ischemic' OR 'ischemic post-conditioning' OR 'ischemic post conditioning' OR 'post-conditioning, ischemic' OR 'ischemic conditioning'/exp OR 'ischemic conditioning' OR 'ischemic postconditioning'/exp OR 'ischemic postconditioning' |
| #3 | #1 OR #2 |
| #4 | 'Kidney Transplantation'/exp |
| #5 | 'Kidney Transplantation' OR 'Renal Transplantation' OR 'Renal Transplantations' OR 'Transplantations, Renal' OR 'Transplantation, Renal' OR 'Grafting, Kidney' OR 'Kidney Grafting' OR 'Transplantation, Kidney' OR 'Kidney Transplantations' OR 'Transplantations, Kidney' |
| #6 | #4 OR #5 |
| #7 | #3 AND #6 |

**Cochrane Library**

| **Search** | **Query** |
| --- | --- |
| #1 | MeSH descriptor: [Ischemic Preconditioning] explode all trees |
| #2 | (preconditioning, ischemic) OR (ischemic pre-conditioning) OR (ischemic pre conditioning) OR (pre-conditioning, ischemic) OR (remote ischemic preconditioning) OR (ischaemic preconditioning) OR (remote ischaemic preconditioning) OR (RIPC) |
| #3 | #1 OR #2 |
| #4 | MeSH descriptor: [Ischemic Postconditioning] explode all trees |
| #5 | (Postconditioning, Ischemic) OR (Ischemic Post-Conditioning) OR (Ischemic Post Conditioning) OR (Post-Conditioning, Ischemic) OR (Ischemic conditioning) |
| #6 | #4 OR #5 |
| #7 | #6 OR #3 |
| #8 | MeSH descriptor: [Kidney Transplantation] explode all trees |
| #9 | (Kidney Transplantation) OR (Renal Transplantation) OR (Renal Transplantations) OR (Transplantations, Renal) OR (Transplantation, Renal) OR (Grafting, Kidney) OR (Kidney Grafting) OR (Transplantation, Kidney) OR (Kidney Transplantations) OR (Transplantations, Kidney) |
| #10 | #8 OR #9 |
| #11 | #10 AND #7 |

**Supplementary Figures**

**
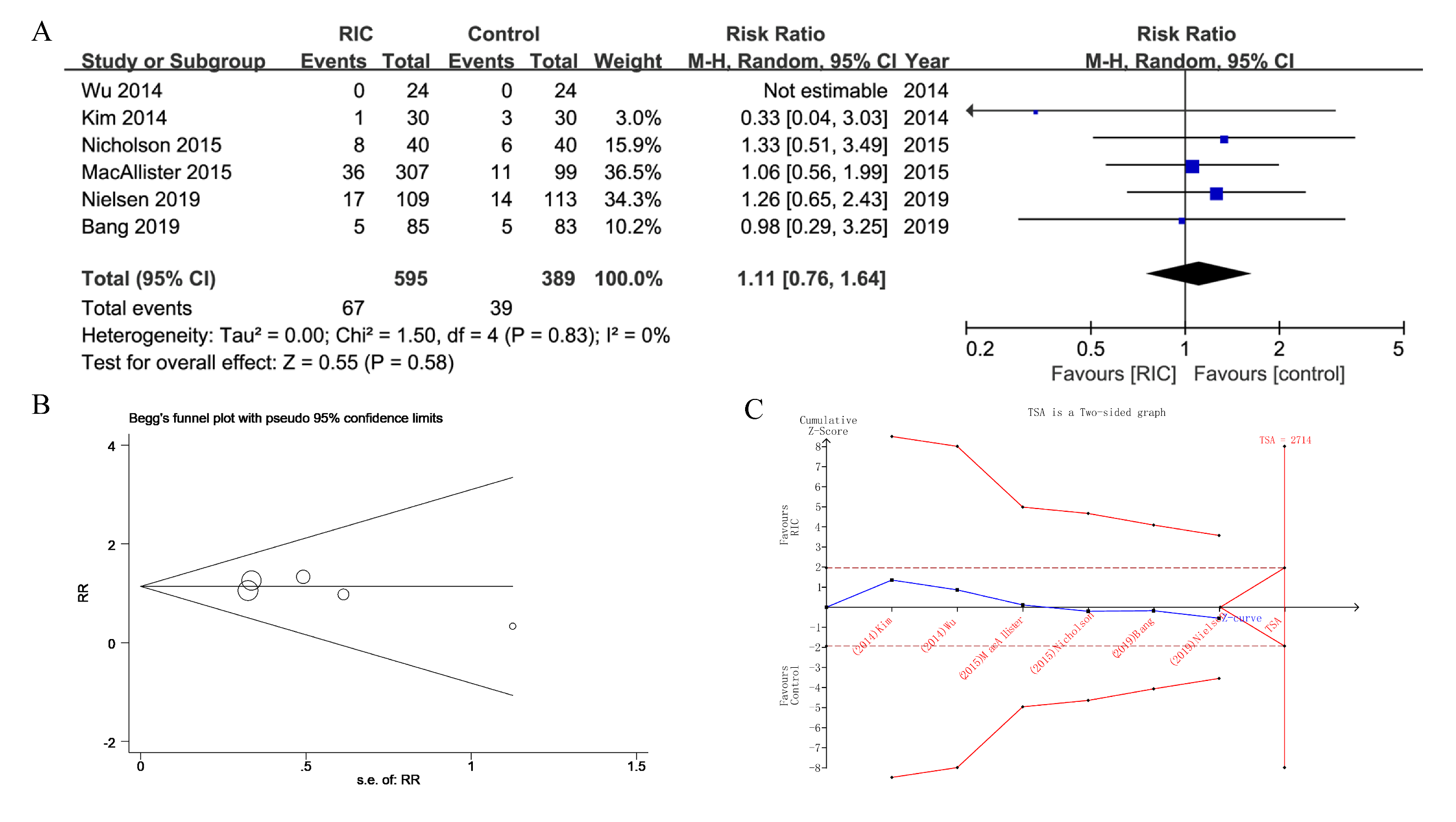
**

**Supplementary Figure 1.** Pooled result of AR in patients undergoing kidney transplantation between RIC and control. (A) forest plot; (B) Begg’s funnel plot; (C) TSA diagram. Analysis is based on a relative risk reduction (RRR) of 30% and a control event rate of 10%. The inward sloping red lines indicate the trial sequential monitoring boundary, the outward sloping red lines indicate the futility boundary; brown lines indicate the conventional benefit boundary; blue line is the Z-curve; SD, standard deviation; CI, confidence interval; M-H, Mantel-Haenszel; RIC, remote ischemic conditioning; RR, risk ratio; s.e., standard error; TSA, Trial sequential analysis; RIS, Required information size.

**
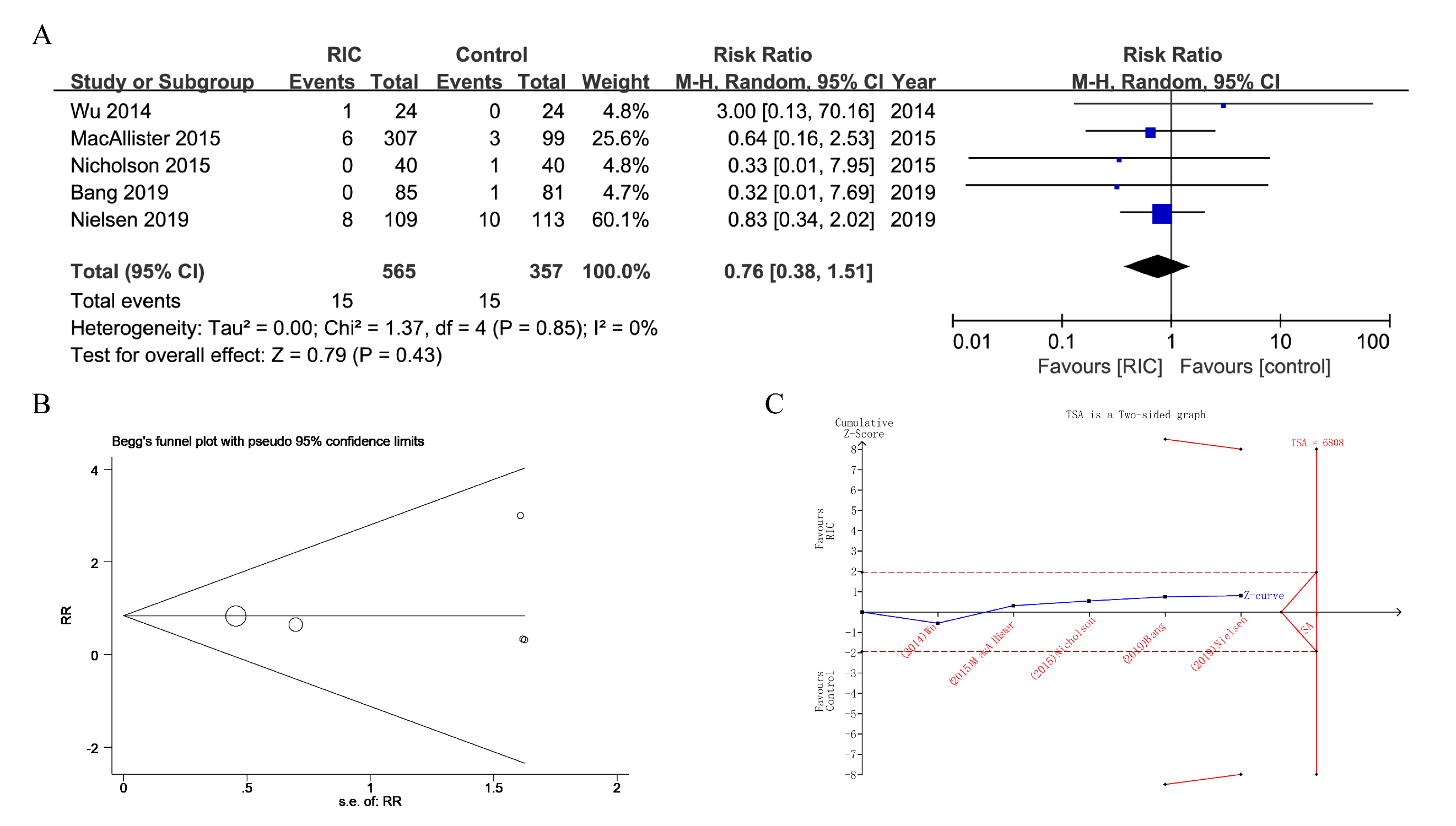
**

**Supplementary Figure 2.** Pooled result of graft loss in patients undergoing kidney transplantation between RIC and control. (A) forest plot; (B) Begg’s funnel plot; (C) TSA diagram. Analysis is based on a relative risk reduction (RRR) of 30% and a control event rate of 4.2%. The inward sloping red lines indicate the trial sequential monitoring boundary, the outward sloping red lines indicate the futility boundary; brown lines indicate the conventional benefit boundary; blue line is the Z-curve; SD, standard deviation; CI, confidence interval; M-H, Mantel-Haenszel; RIC, remote ischemic conditioning; RR, risk ratio; s.e., standard error; TSA, Trial sequential analysis; RIS, Required information size.


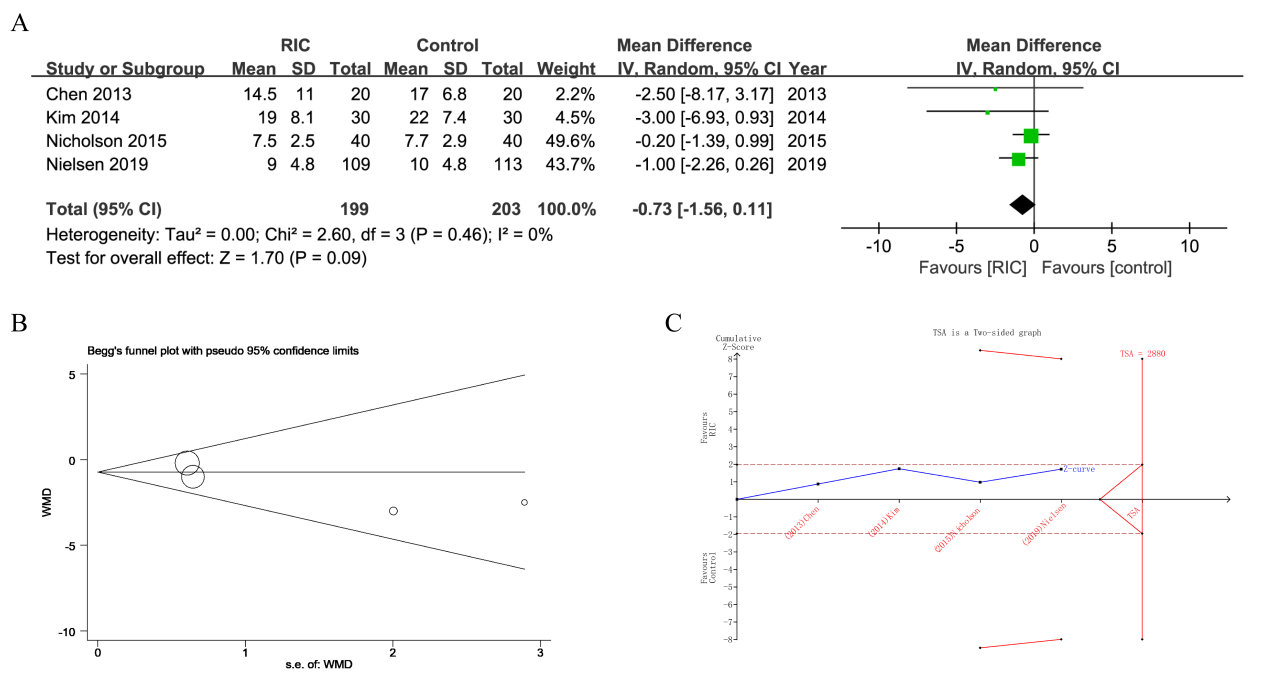


**Supplementary Figure 3.** Pooled result of hospital stay in patents undergoing kidney transplantation between RIC and control. (A) forest plot; (B) Begg’s funnel plot; (C) TSA diagram. Analysis is based on a power of 90%. The inward sloping red lines indicate the trial sequential monitoring boundary, the outward sloping red lines indicate the futility boundary; brown lines indicate the conventional benefit boundary; blue line is the Z-curve; SD, standard deviation; CI, confidence interval; M-H, Mantel-Haenszel; RIC, remote ischemic conditioning; WMD, weighted mean difference; s.e., standard error; TSA, Trial sequential analysis; RIS, Required information size.

**
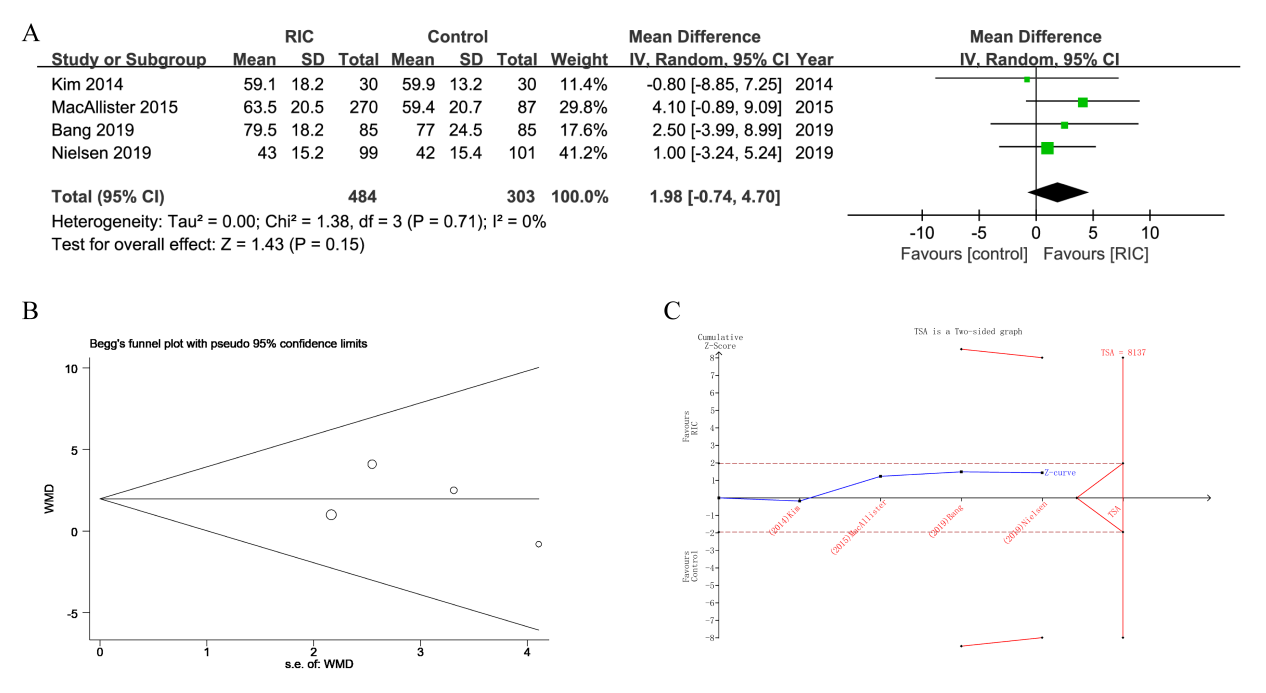
Supplementary Figure 4.** Pooled result of eGFR at 12 months in patients undergoing kidney transplantation between RIC and control. (A) forest plot; (B) Begg’s funnel plot; (C) TSA diagram. Analysis is based on a power of 90%. The inward sloping red lines indicate the trial sequential monitoring boundary, the outward sloping red lines indicate the futility boundary; brown lines indicate the conventional benefit boundary; blue line is the Z-curve; SD, standard deviation; CI, confidence interval; M-H, Mantel-Haenszel; RIC, remote ischemic conditioning; WMD, weighted mean difference; s.e., standard error; TSA, Trial sequential analysis; RIS, Required information size.


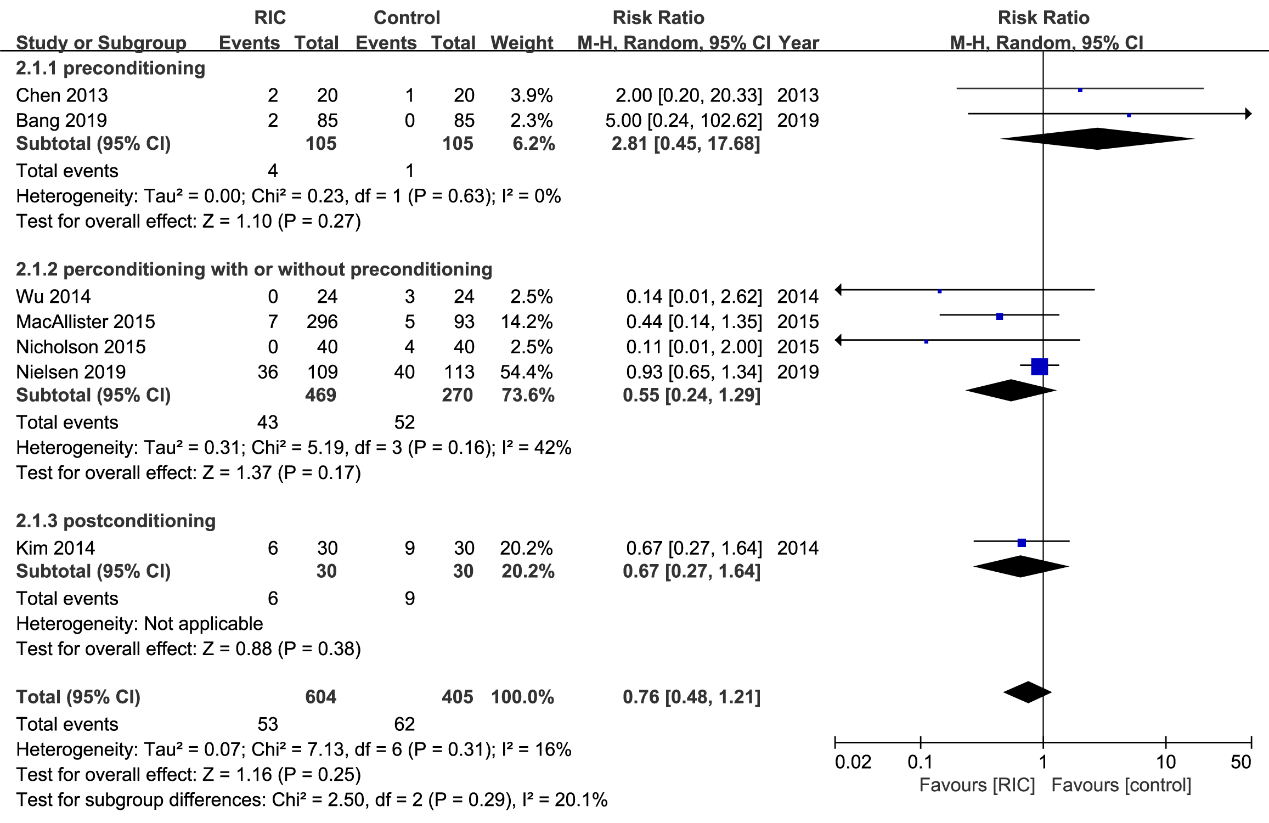


**Supplementary Figure 5.** Subgroup analysis for DGF based on three RIC types. CI, confidence interval; M-H, Mantel-Haenszel; RIC, remote ischemic conditioning.


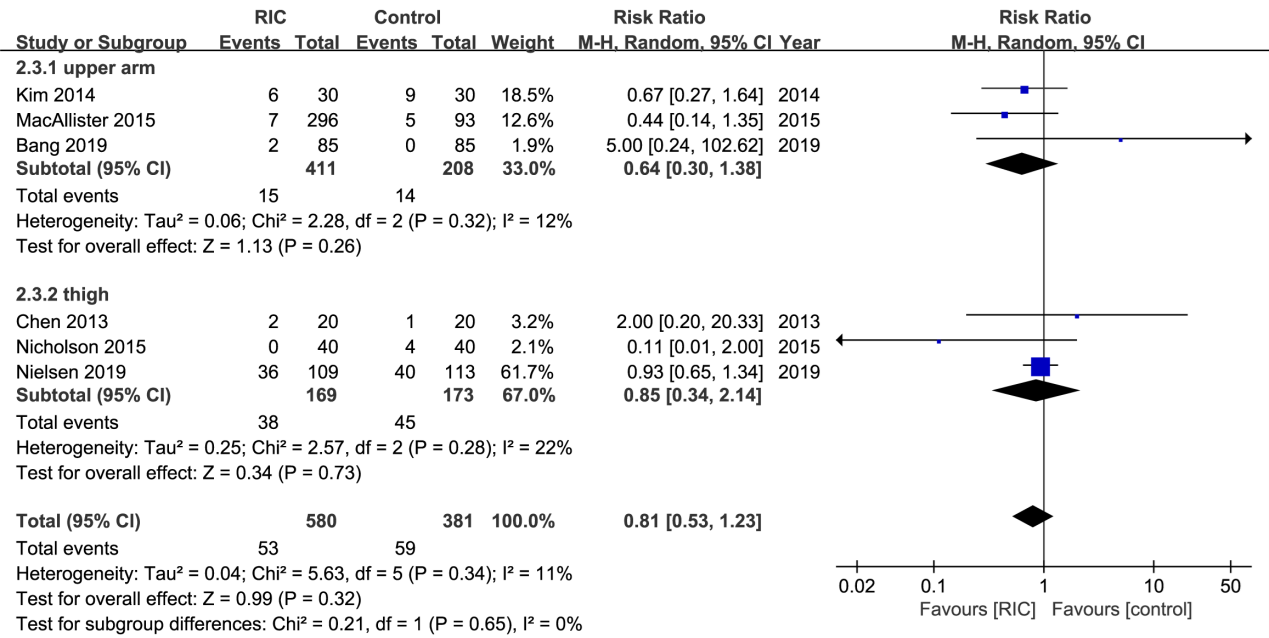


**Supplementary Figure 6.** Subgroup analysis for DGF based on RIC imposed at upper arm versus thigh. CI, confidence interval; M-H, Mantel-Haenszel; RIC, remote ischemic conditioning.


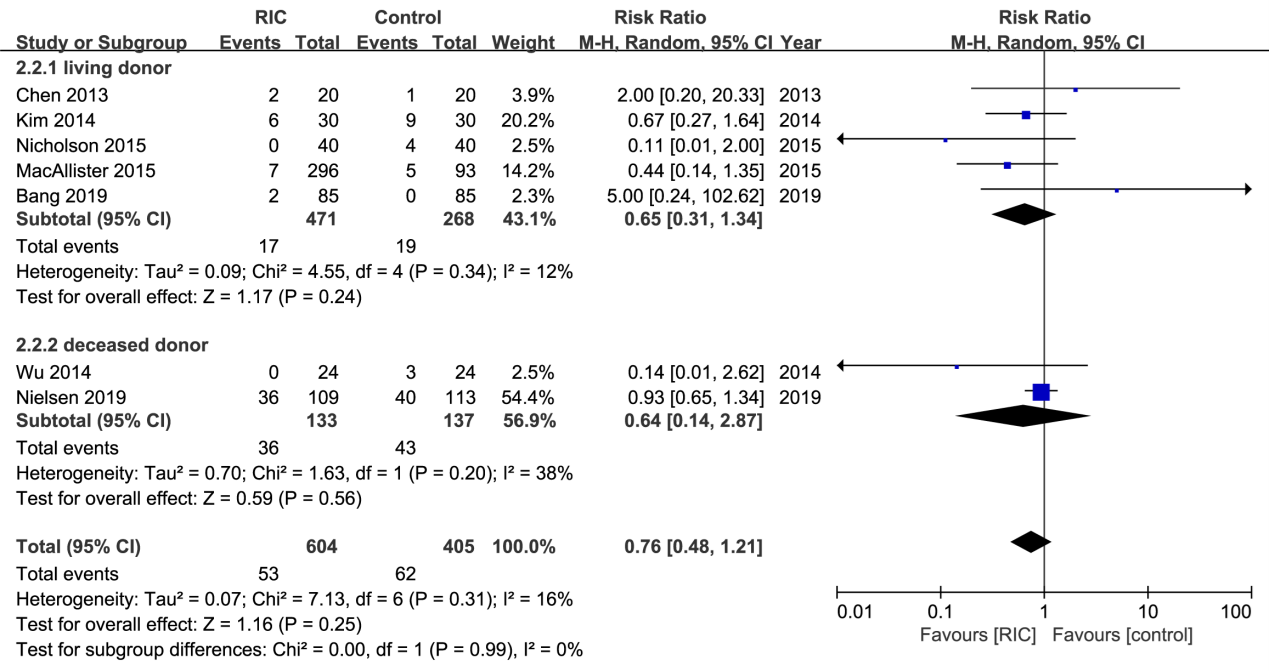
**Supplementary Figure 7.** Subgroup analysis for DGF based on living donor versus deceased donor. CI, confidence interval; M-H, Mantel-Haenszel; RIC, remote ischemic conditioning.


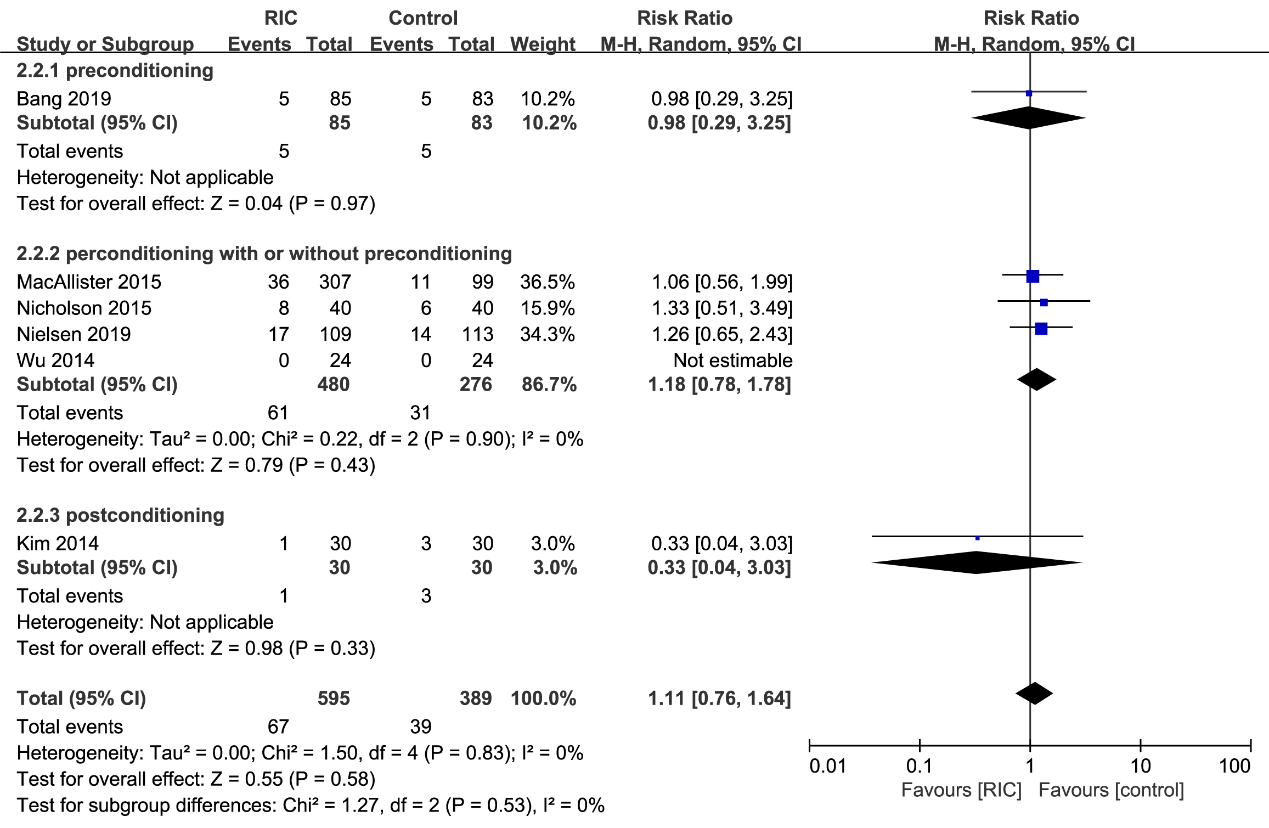
 **Supplementary Figure 8.** Subgroup analysis for AR based on three RIC types. CI, confidence interval; M-H, Mantel-Haenszel; RIC, remote ischemic conditioning.


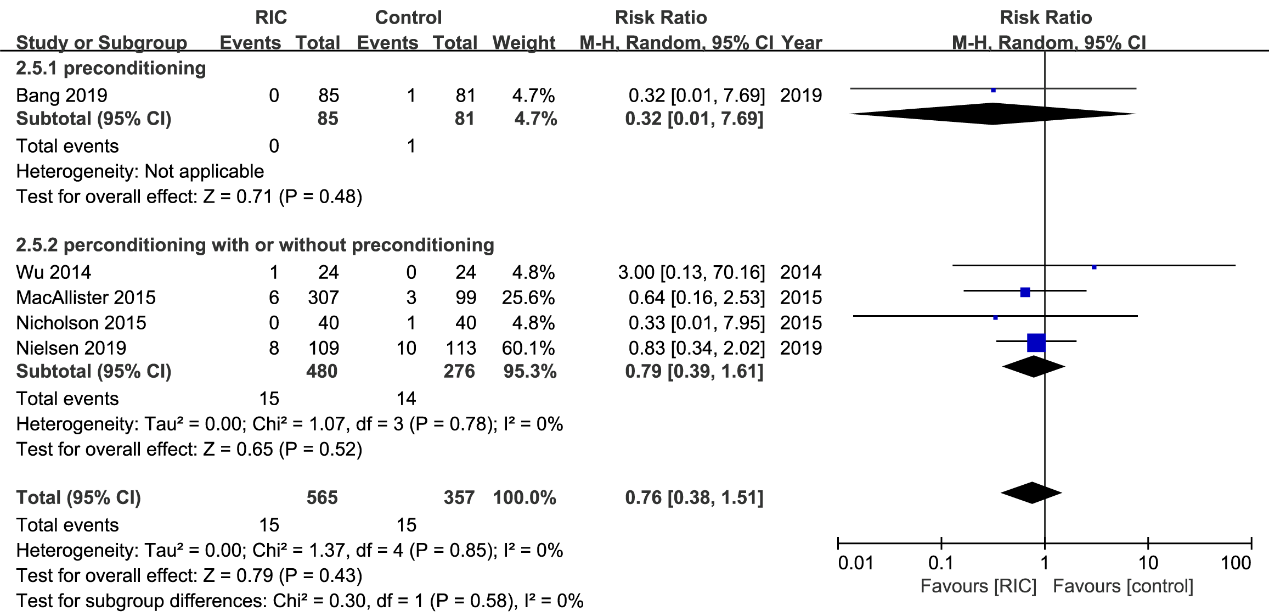
**Supplementary Figure 9.** Subgroup analysis for graft loss based on three RIC types. CI, confidence interval; M-H, Mantel-Haenszel; RIC, remote ischemic conditioning.


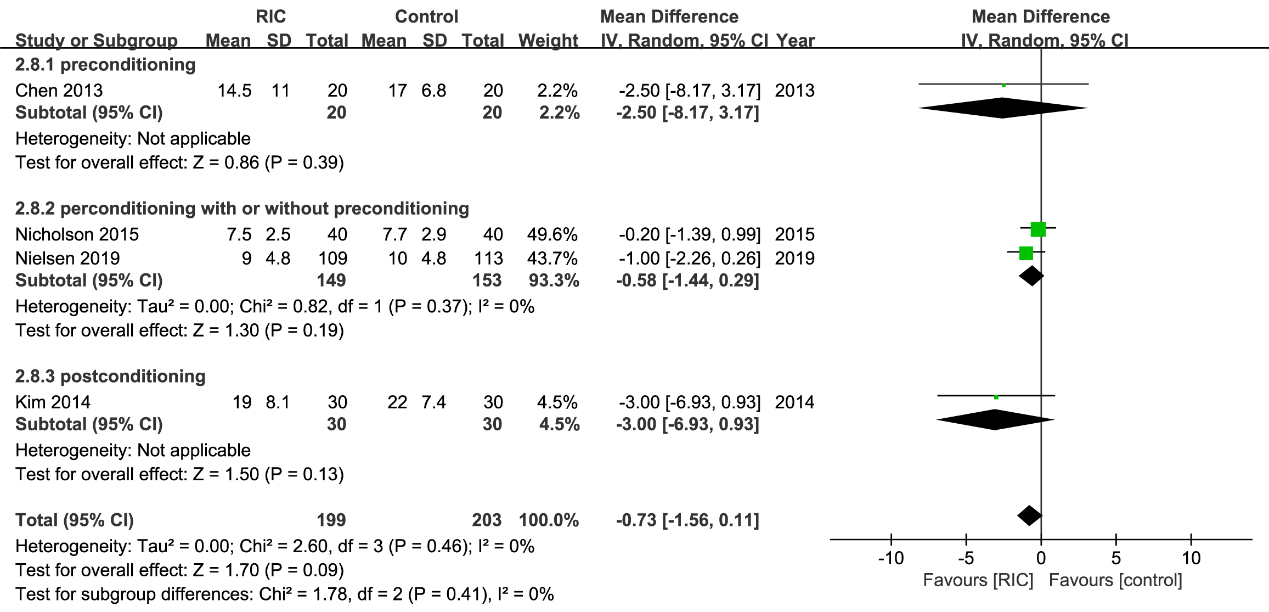
**Supplementary Figure 10.** Subgroup analysis for hospital stay based on three RIC types. CI, confidence interval; M-H, Mantel-Haenszel; RIC, remote ischemic conditioning.


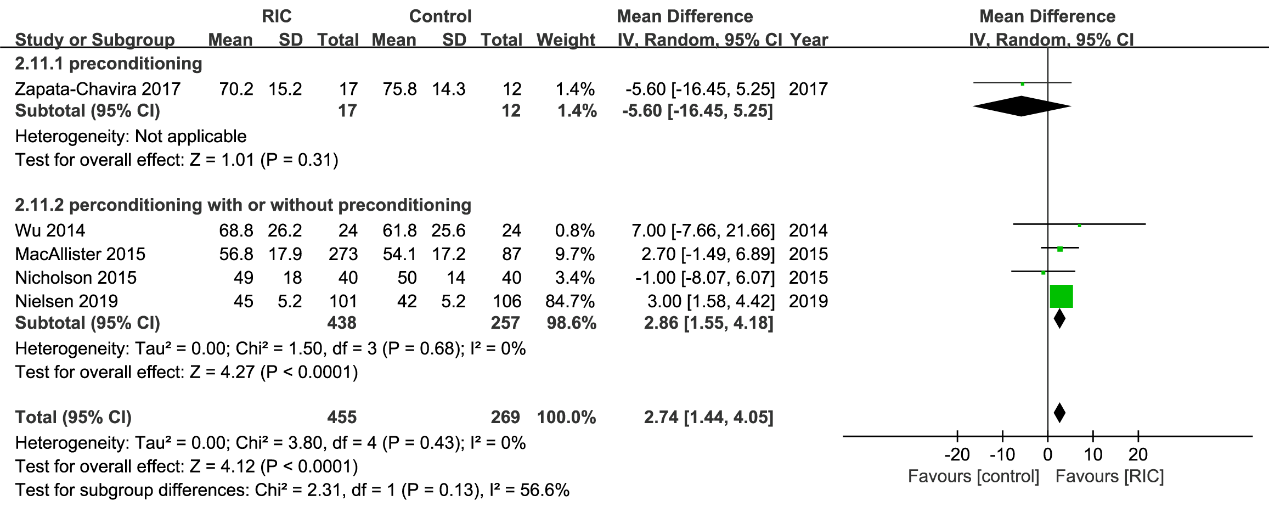
**Supplementary Figure 11.** Subgroup analysis for eGFR at 3 months based on three RIC types. CI, confidence interval; M-H, Mantel-Haenszel; RIC, remote ischemic conditioning.


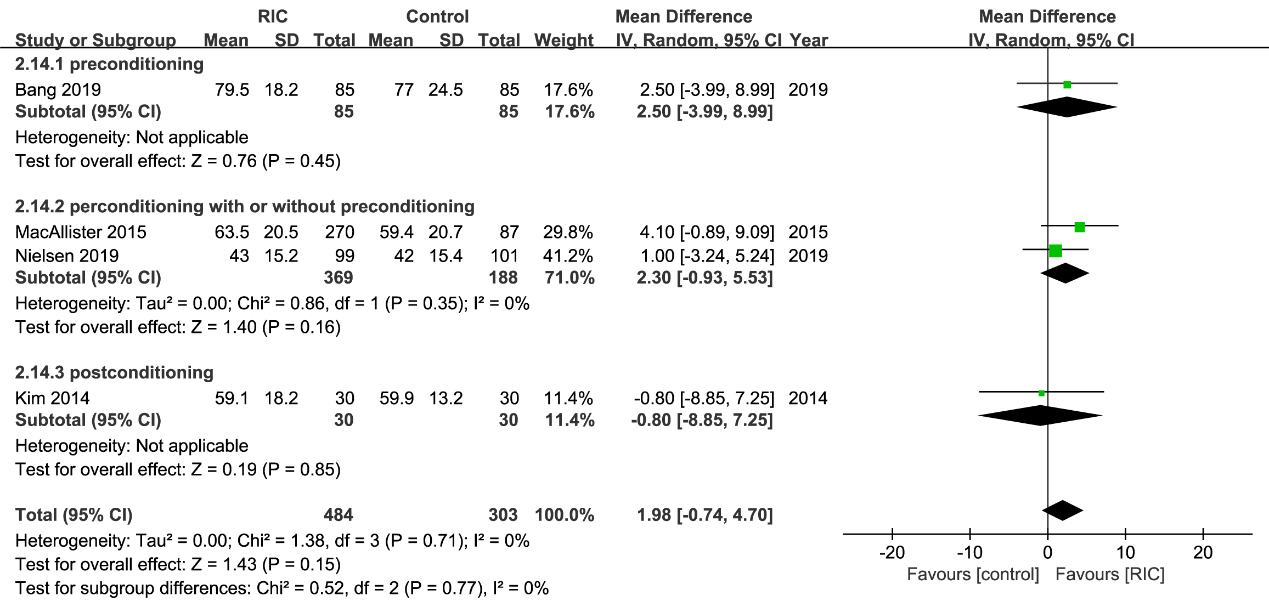
**Supplementary Figure 12.** Subgroup analysis for eGFR at 12 months based on three RIC types. CI, confidence interval; M-H, Mantel-Haenszel; RIC, remote ischemic conditioning.


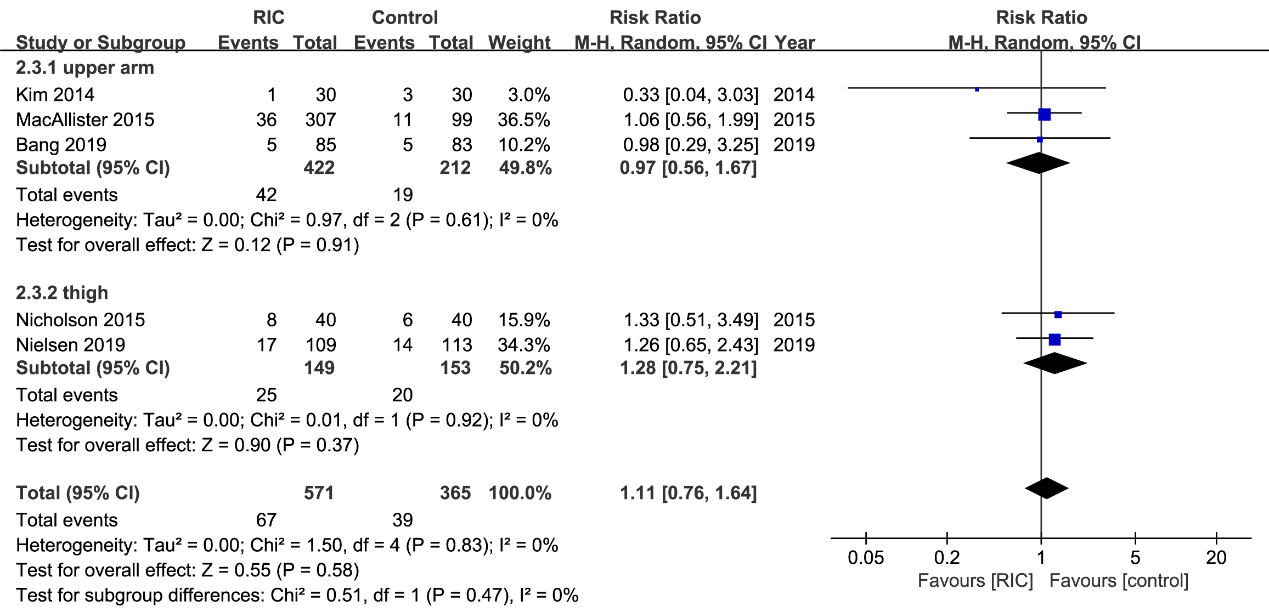
**Supplementary Figure 13.** Subgroup analysis for AR based on RIC imposed at upper arm versus thigh. CI, confidence interval; M-H, Mantel-Haenszel; RIC, remote ischemic conditioning.


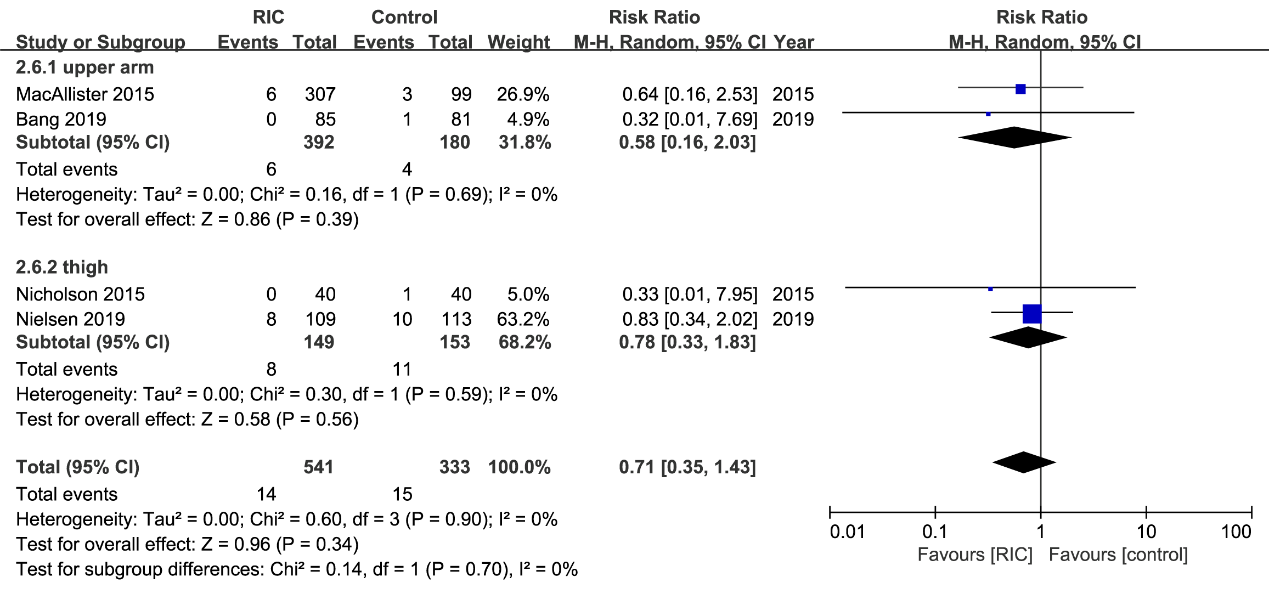
**Supplementary Figure 14.** Subgroup analysis for graft loss based on RIC imposed at upper arm versus thigh. CI, confidence interval; M-H, Mantel-Haenszel; RIC, remote ischemic conditioning.


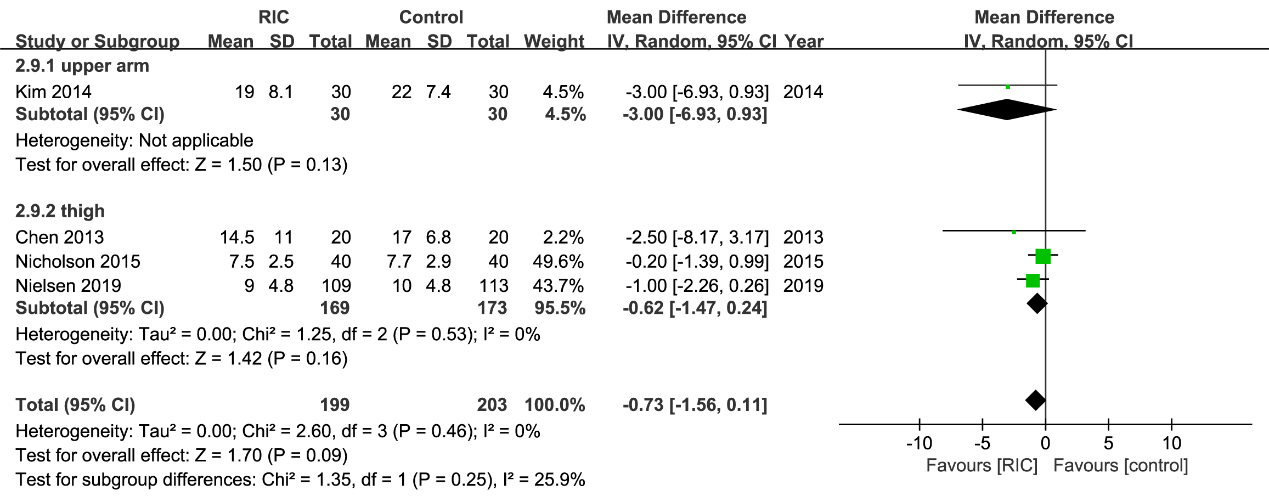
**Supplementary Figure 15.** Subgroup analysis for hospital stay based on RIC imposed at upper arm versus thigh. CI, confidence interval; M-H, Mantel-Haenszel; RIC, remote ischemic conditioning.


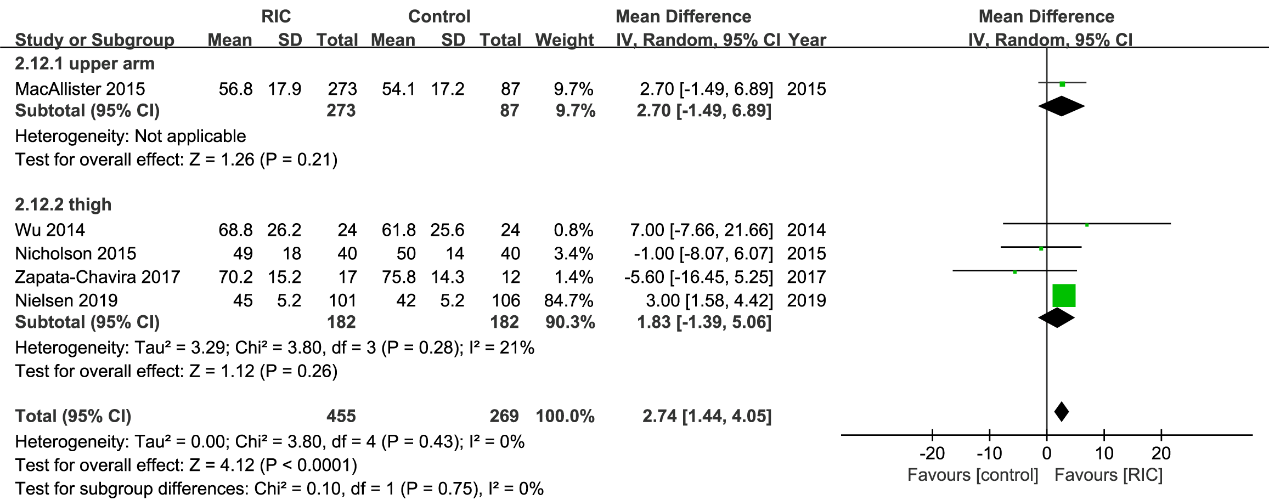
**Supplementary Figure 16.** Subgroup analysis for eGFR at 3 months based on RIC imposed at upper arm versus thigh. CI, confidence interval; M-H, Mantel-Haenszel; RIC, remote ischemic conditioning.


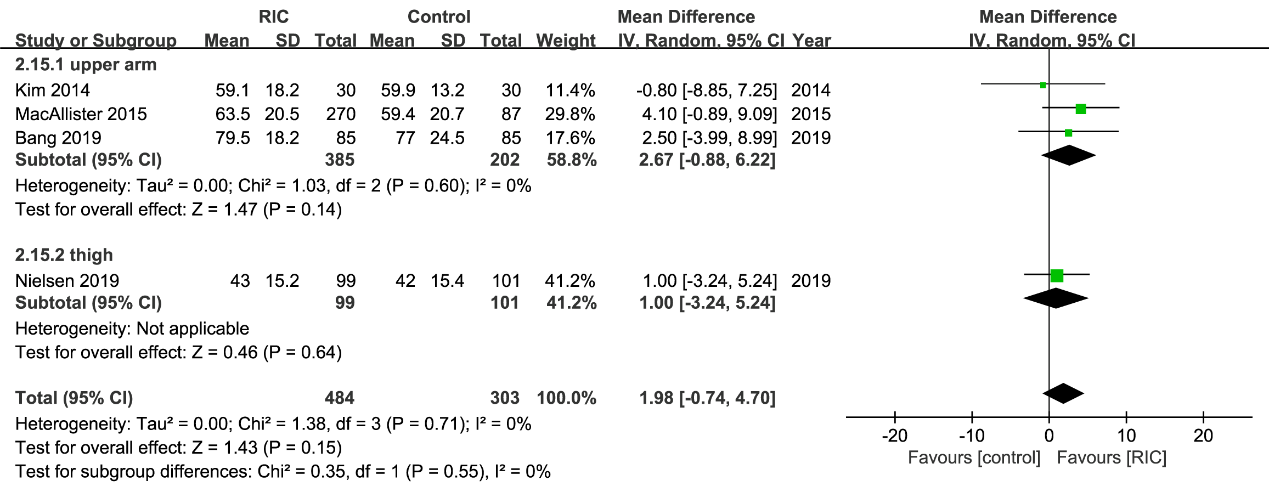
**Supplementary Figure 17.** Subgroup analysis for eGFR at 12 months based on RIC imposed at upper arm versus thigh. CI, confidence interval; M-H, Mantel-Haenszel; RIC, remote ischemic conditioning.


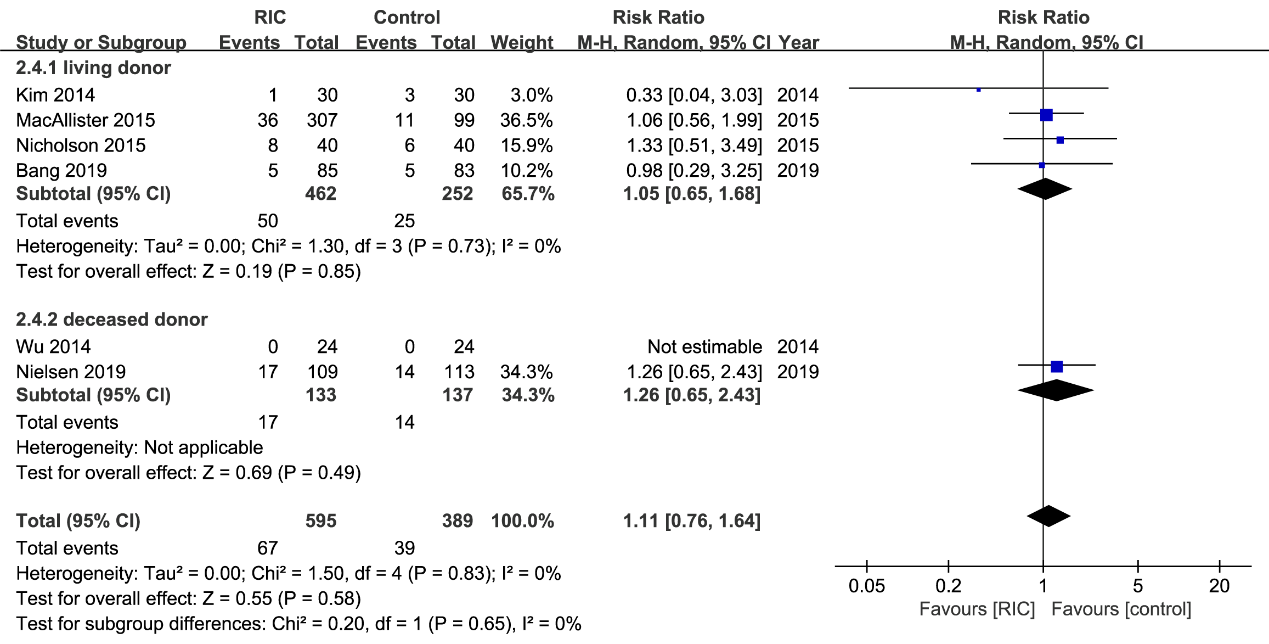
**Supplementary Figure 18.** Subgroup analysis for AR based on living donor versus deceased donor. CI, confidence interval; M-H, Mantel-Haenszel; RIC, remote ischemic conditioning.


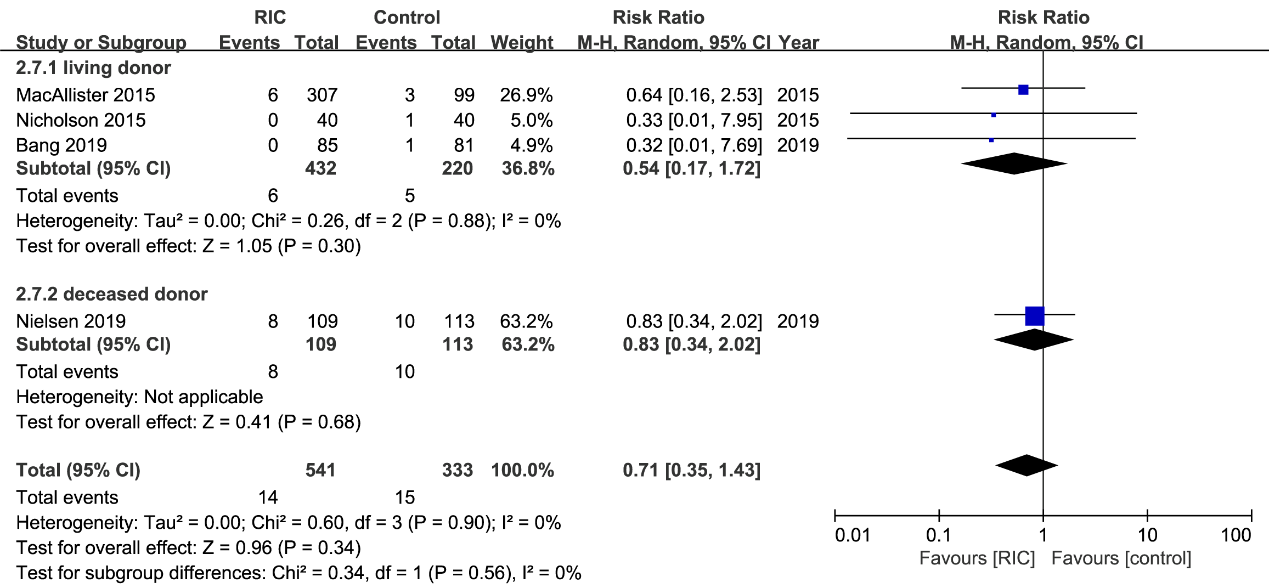
**Supplementary Figure 19.** Subgroup analysis for graft loss based on living donor versus deceased donor. CI, confidence interval; M-H, Mantel-Haenszel; RIC, remote ischemic conditioning.


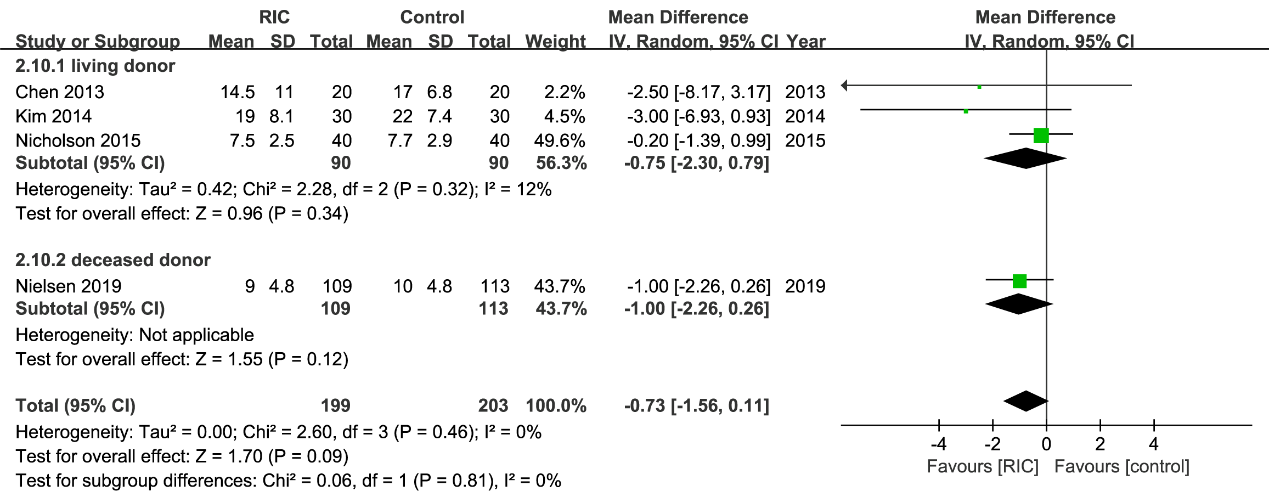
**Supplementary Figure 20.** Subgroup analysis for hospital stay based on living donor versus deceased donor. CI, confidence interval; M-H, Mantel-Haenszel; RIC, remote ischemic conditioning.


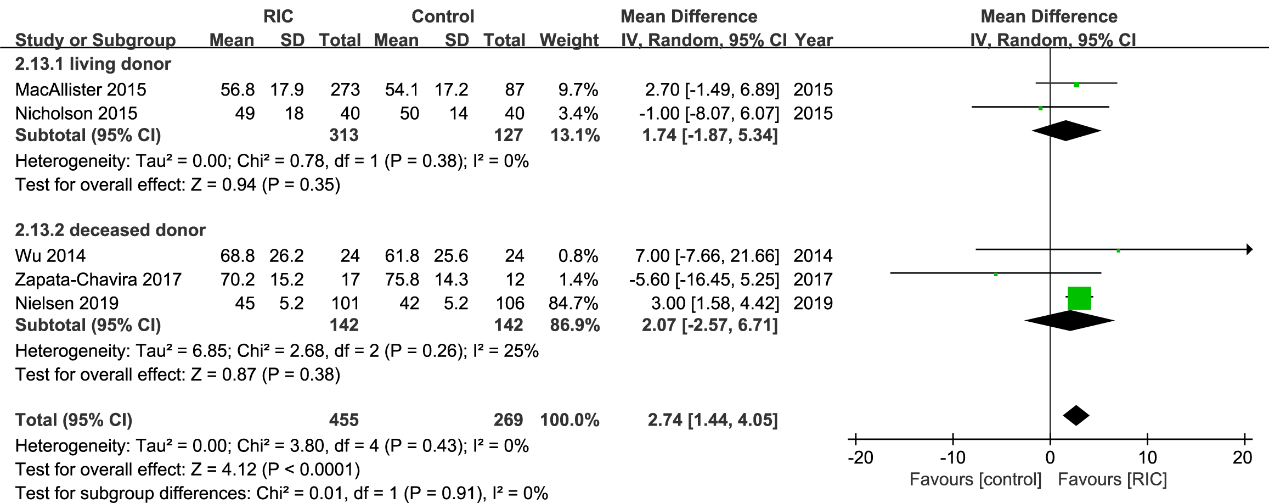
**Supplementary Figure 21.** Subgroup analysis for eGFR at 3 months based on living donor versus deceased donor. CI, confidence interval; M-H, Mantel-Haenszel; RIC, remote ischemic conditioning.


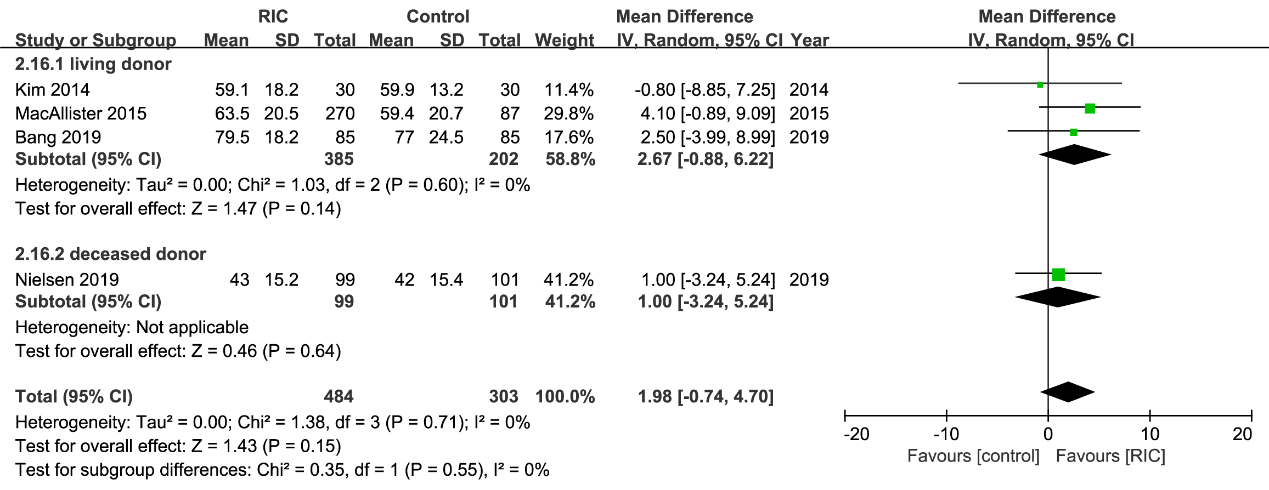
**Supplementary Figure 22.** Subgroup analysis for eGFR at 12 months based on living donor versus deceased donor. CI, confidence interval; M-H, Mantel-Haenszel; RIC, remote ischemic conditioning.
